# Soluble IL-2 receptor and memory Treg profiles differentiate early rheumatoid arthritis from undifferentiated arthritis at initial presentation

**DOI:** 10.3389/fimmu.2026.1844268

**Published:** 2026-06-09

**Authors:** Xiaoyu Zi, Yifang Shi, Yicong Zhao, Chong Gao, Caihong Wang

**Affiliations:** 1 Department of Rheumatology, The Second Hospital of Shanxi Medical University, Taiyuan, Shanxi, China; 2 Shanxi Key Laboratory of Rheumatism Immune Microecology, Taiyuan, Shanxi, China; 3 Shanxi Precision Medical Engineering Research Center for Rheumatology, Taiyuan, Shanxi, China; 4 Pathology, Joint Program in Transfusion Medicine, Brigham and Women’s Hospital/Children’s Hospital, Harvard Medical School, Boston, MA, United States

**Keywords:** immune signature, memory regulatory T cells, rheumatoid arthritis, Th17/Treg imbalance, undifferentiated arthritis

## Abstract

**Background:**

Rheumatoid arthritis (RA) is a significant global health issue. Early diagnosis remains clinically challenging, particularly in patients with inflammatory arthritis who do not yet fulfill 2010 ACR/EULAR classification criteria (undifferentiated arthritis, UA).

**Methods:**

This retrospective study enrolled 83 treatment-experienced RA (TE-RA), 49 treatment-naïve RA (TN-RA), and 51 seropositive UA patients, as well as 60 healthy controls. Peripheral lymphocyte subsets and serum cytokines were profiled using flow cytometry and bead arrays. Least absolute shrinkage and selection operator (LASSO) regression was utilized to develop an immune-based derivation-stage classifier for distinguishing TN-RA from UA, with internal validation by bootstrap resampling (B = 1000).

**Results:**

Immunophenotypic analysis identified distinct immune profiles between TN-RA and UA at initial presentation. TN-RA was characterized by IL-2 signaling exhaustion (elevated sIL-2R, decreased IL-2), systemic inflammation (elevated IL-6, IFN-γ, and TNF-α), and compensatory memory Treg expansion (increased CD45RO+ Tregs). In contrast, UA exhibited a Th17/Treg imbalance with relatively preserved Th2 and Th17 responses. An eight-feature immune signature (sIL-2R, IL-6, CD45RO+ Tregs, CD45RO+ Treg%, IFN-γ, Th17/Treg ratio, Th2, Th17%) discriminated TN-RA from UA with an optimism-corrected AUC of 0.959 (95% CI: 0.923–0.995), adjusted for age, sex, BMI, and disease duration. sIL-2R and IL-6 were the strongest contributors, consistent with their central roles in RA pathophysiology.

**Conclusions:**

In the present study, an immune-based derivation-stage classifier showed potential for distinguishing TN-RA from UA at initial presentation. The IL-2-Treg axis perturbation represents a potential pathophysiological distinction, with sIL-2R as a candidate biomarker. These findings suggest that objective immune profiling may inform clinical decision-making when conventional criteria are inconclusive.

## 1 Introduction

Rheumatoid arthritis (RA) is a chronic autoimmune disorder that causes synovitis, damaging cartilage and bone structures (1, 2). RA is prevalent worldwide, with prevalence rates varying from 0.24% to 2% (3, 4). The most prominent manifestation of RA is its impact on the joints, but it can also affect nearly every other organ system in the body (5). The chronic and disabling features of RA carry a substantial burden for individuals and society (1); therefore, an early diagnosis and suitable intervention are crucial to control inflammation and limit damage (6). Nonetheless, early diagnosis is difficult due to the ambiguous initial symptoms and the complicated, heterogeneous immunological dysregulation that characterizes the disease, especially in the preclinical or early undifferentiated stage.

The invasion of diverse immune cells and the disruption of the cytokine network within the synovium collectively induce chronic inflammation and joint destruction in RA (7). The equilibrium among CD4+ T cells is crucial for maintaining immunological homeostasis and preventing the emergence of autoimmune disorders (8). The imbalance among CD4+ T cell subsets plays a critical role in the pathogenesis of RA. Beyond the classic T helper 1 (Th1)/Th2 imbalance, significant breakthroughs have been made regarding the imbalance between Th17 cells and regulatory T cells (Tregs) (7). In RA, Th17 cells drive synovial inflammation through interleukin-17 (IL-17)-mediated induction of pro-inflammatory cytokines [tumor necrosis factor-α (TNF-α), IL-6, and IL-1β], chemokines, and matrix metalloproteinases, leading to cartilage degradation and bone erosion (9). Conversely, Tregs facilitate the anti-inflammatory response by the secretion of IL-10 and transforming growth factor-β (TGF-β), therefore sustaining autoimmune tolerance (10). Tregs exhibit phenotypic heterogeneity based on CD45 isoform expression. CD45RA+ Tregs represent a naïve or resting population that has not encountered a cognate antigen, whereas CD45RO+ Tregs constitute an antigen-experienced memory subset with enhanced migratory capacity and sustained suppressive function (11). This phenotypic distinction carries clinical relevance in RA: the shift from CD45RA+ to CD45RO+ Tregs reflects chronic antigenic stimulation, and the relative expansion of memory Tregs in established disease may represent a compensatory yet functionally inadequate regulatory response (12). The Th17/Treg imbalance is considered a critical feature driving RA pathogenesis. Primary studies have demonstrated elevated Th17 and diminished functional Treg proportions in the peripheral blood of RA patients, and the Th17/Treg ratio correlates positively with disease activity scores (13, 14). Notably, IL-2 signaling sits at the nexus of this balance. IL-2 is indispensable for Treg development, survival, and suppressive function through activation of the JAK1-STAT5 pathway and maintenance of Foxp3 expression (15). Tregs express the high-affinity IL-2 receptor α-chain (CD25) and depend on exogenous IL-2, as they cannot autonomously produce this cytokine. In RA, diminished circulating IL-2 coupled with elevated soluble IL-2 receptor (sIL-2R), a marker of T-cell activation and IL-2 consumption, suggests dysregulated IL-2 homeostasis that may compromise Treg competence (16). This IL-2–Treg axis perturbation provides a mechanistic framework for understanding the transition from inflammatory dysregulation to failed immune tolerance in established RA.

Seropositive undifferentiated arthritis (UA) occupies a critical position in the RA disease continuum, representing the clinical stage between systemic autoimmunity and fulfilled RA (17). The 2010 American College of Rheumatology and the European League Against Rheumatism (ACR/EULAR) classification criteria create a diagnostic gap: autoantibody-positive patients with inflammatory arthritis who do not fully meet criteria are labeled UA, a heterogeneous category that includes early RA. This ambiguity delays or obscures treatment decisions, underscoring the urgent need for biomarkers that distinguish RA from UA at first presentation. The pre-RA phase, characterized by circulating autoantibodies without clinical arthritis, has emerged as a fertile ground for biomarker discovery. Prospective studies established that anti-cyclic citrullinated peptide (anti-CCP) antibodies and rheumatoid factor (RF) precede clinical onset by several years in a substantial proportion of patients (18, 19). More recently, there is research reported that progression to RA in at-risk individuals is characterized by systemic inflammation alongside T- and B-cell dysregulation, underscoring the immune perturbations that precede clinical disease onset (20). Therefore, the UA-to-RA transition represents a clinically actionable interval where immune phenotyping could guide immediate therapeutic decisions. Previous efforts to stratify UA patients have leveraged synovial imaging, multiplex cytokine arrays, and genetic risk scores. Magnetic resonance imaging of the hand and foot detects subclinical inflammation in clinically suspect arthralgia and predicts progression to RA (21), while bone marrow edema on MRI independently predicts RA development in early undifferentiated arthritis (22). While these imaging-based approaches improve risk stratification, they do not provide an objective immune signature for differentiating concurrent RA from UA at initial presentation.

To meet this clinical requirement, we initially conducted a comprehensive comparative analysis of immunophenotypic profiles across the UA, RA, and healthy control (HC) groups. Subsequently, by comparing treatment-naïve (TN-RA) (fulfilling 2010 ACR/EULAR criteria) with UA (not fulfilling criteria despite similar clinical features) at the same initial presentation time point and strictly excluding RA patients exposed to immunomodulatory therapies, we aimed to (i) characterize qualitative immune landscape remodeling through principal component analysis (PCA); (ii) develop and validate an objective immune-based derivation-stage classifier using least absolute shrinkage and selection operator (LASSO) regression with bootstrap validation; and (iii) identify potential pathophysiological mechanisms—IL-2 signaling exhaustion and compensatory memory Treg expansion versus Th17-predominant inflammation distinguishes these conditions biologically when clinical differentiation proves challenging. This study thereby establishes an objective, immune signature-based derivation-stage framework to enhance clinical decision-making and reduce diagnostic delay at the critical first encounter.

## 2 Materials and methods

### 2.1 Clinical patients and study design

This retrospective study included 132 patients with RA [49 TN-RA and 83 treatment-experienced (TE-RA)] and 51 patients with seropositive UA, all recruited from the Rheumatology Department of the Second Hospital of Shanxi Medical University between July 2024 and July 2025. All diagnoses of RA adhered to the 2010 revised classification criteria set forth by the ACR/EULAR. The TN-RA group did not take glucocorticoids or disease-modifying antirheumatic medications (DMARDs) and had a disease duration longer than six months. Seropositive UA is a diagnosis of exclusion characterized by elevated serum anti-CCP antibodies, with or without increased RF, in patients exhibiting confirmed arthritis who do not meet the 2010 ACR/EULAR diagnostic criteria for RA or other specific arthritic conditions (23, 24). Individuals with other autoimmune disorders, cancers, significant infections, pregnancy, or undergoing low-dose human recombinant IL-2 treatment were excluded. Furthermore, 60 healthy individuals, matched for age and sex with RA patients, were selected from the health assessment center of our hospital. Blood samples for all clinical parameters were obtained on the morning of the consultation following an overnight fast. The Ethics Committee of the Second Hospital of Shanxi Medical University approved this study [Approval No. (2019) YX (105)].

This study utilized a case-control design to compare two unique patient groups at initial presentation: patients who met the 2010 ACR/EULAR RA criteria (TN-RA) and those with analogous clinical characteristics who did not fulfill the criteria (UA). Both groups were strictly treatment-naïve to guarantee that the observed immunophenotypic variations represent inherent disease biology rather than the effects of medication.

### 2.2 Clinical and laboratory data collection

We gathered clinical and laboratory data for all RA and UA patients. The collected data encompassed patient gender, age, body mass index (BMI), disease duration, lifestyle variables, and medication use. The serological markers assessed comprised erythrocyte sedimentation rate (ESR), C-reactive protein (CRP), complete blood count, coagulation profile, liver function tests, renal function tests, calcium levels, 25-hydroxyvitamin D, anti-perinuclear factor, antinuclear antibodies, RF, anti-mutated citrullinated vimentin (anti-MCV), and anti-CCP antibodies. The disease activity in rheumatoid arthritis patients was evaluated with the Disease Activity Score 28-joint count (DAS28)-ESR.

### 2.3 Flow cytometry

**Figure 1:**
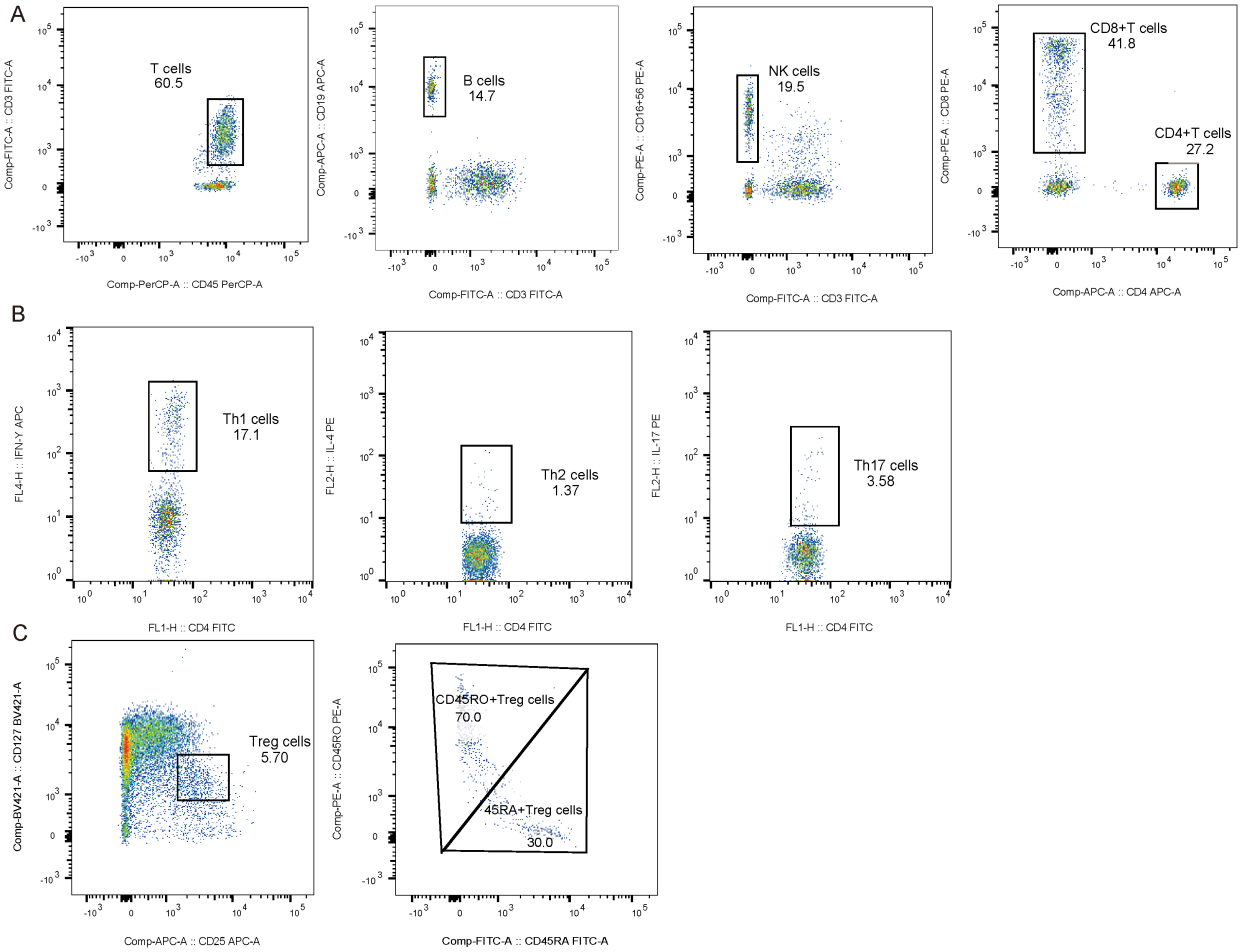
Representative flow cytometric staining of target immune populations. **(A)** Peripheral blood lymphocyte subsets were identified as T cells (CD45+CD3+), B cells (CD45+CD3−CD19+), NK cells (CD45+CD3−CD16+CD56+), CD4+ T cells (CD45+CD3+CD4+), and CD8+ T cells (CD45+CD3+CD8+). **(B)** CD4+ T cell subsets: Th1 (CD4+IFN-γ+), Th2 (CD4+IL-4+), and Th17 (CD4+IL-17A+). **(C)** Regulatory T cells were gated as CD4+CD25+CD127low/−, with further subdivision into CD45RA+ naïve Tregs and CD45RO+ memory Tregs.

We employed flow cytometry to analyze distinct lymphocyte populations, including CD4+ T cells and T regulatory cells, in peripheral blood. Two milliliters of heparin-anticoagulated peripheral blood were collected and subjected to immunophenotyping with fluorochrome-conjugated monoclonal antibodies (FITC, PE, PerCP, BV421, APC; BD Biosciences). Doublets were excluded based on FSC-A versus FSC-H discrimination. All samples were processed immediately after collection and analyzed within 24 hours under standardized conditions. The complete gating strategy is provided in **Supplementary Figure 1**. Representative flow cytometric staining of target immune populations is shown in **Figure 1**.

Lymphocytes: Each of the two BD Trucount tubes (A and B) had 50 microliters of blood put into them. Anti-CD3-FITC/CD8-PE/CD45-PerCP/CD4-APC (Cat# 662965) went into tube A, and anti-CD3-FITC/(CD16+CD56)-PE/CD45-PerCP/CD19-APC (Cat# 662965) went into tube B. After 20 minutes of incubation in the dark at 25 °C, 450 μL of 1× FACS hemolysin was added, and the mixture was incubated for another 15 minutes. Cells were washed with phosphate-buffered saline and analyzed within 24 hours. We documented 15,000 occurrences for each sample.

CD4+ T cells: We added PMA, ionomycin, and GolgiStop to 80 μL of whole blood and kept it at 37 °C for 5 hours in a 5% CO_2_ atmosphere. We used anti-CD4-FITC (Cat# 340133) to stain the cells, and then we fixed and permeabilized them. They were then stained with anti-IFN-γ-APC (Cat# 341117) for Th1, anti-IL-4-PE (Cat# 340451) for Th2, and anti-IL-17A-PE (Cat# 560436) for Th17.

Treg cell subsets: After RBC lysis, cells were stained for Tregs using anti-CD4-PerCP (Cat# 340671), CD25-APC (Cat# 340939), and CD127-BV421 (Cat# 562436). We used anti-CD45RA-FITC (Cat# 347723) along with the antibodies we had already mentioned to tag CD45RA+ Tregs. To find CD45RO+ Tregs, we used anti-CD45RO-PE (Cat# 347967).

All procedures followed the manufacturer’s instructions (BD Biosciences). Samples were analyzed using BD Multitest software. To get the absolute counts of CD4+ subsets, we multiplied the subset proportion by the total CD4+ T cell count (cells/μL). The total Treg count was the sum of the absolute counts of CD45RA+ Tregs and CD45RO+ Tregs.

### 2.4 Measurement of cytokine levels

Serum was extracted from 4 ml of venous blood and preserved at -20 °C. Cytokine concentrations were measured by flow cytometry using cytometric bead array kits from Jiangxi Cellgene Biotech Co., Ltd. (Jiangxi, China). The detection ranges were 2.5–2500 pg/mL for IL-2, IL-4, IL-6, IL-10, interferon-γ (IFN-γ), and TNF-α and 10–2500 pg/mL for IL-17. sIL-2R was measured separately with a detection range of 50–7500 U/mL. All procedures followed the manufacturer’s instructions. Samples with cytokine concentrations below the lower limits of detection (LLOD) were assigned a value of LLOD/2 for statistical analysis.

### 2.5 Statistical analysis

Statistical analyses were performed using SPSS (version 23.0; SPSS Inc., Chicago, IL, USA) and R (version 4.5.0). Continuous data exhibiting a normal distribution are expressed as mean ± standard deviation (SD) and evaluated using the independent samples t-test or one-way analysis of variance (ANOVA). Data that do not fit a normal distribution are expressed as the median (interquartile range, IQR) and evaluated using the Mann-Whitney U test or the Kruskal-Wallis H test. We used the chi-square test or Fisher’s exact test, if it was appropriate, to look at categorical variables as numbers (percentages). We utilized Spearman’s rank correlation coefficient to see how the variables were linked. A two-tailed *p*-value lower than 0.05 was deemed statistically significant.

To characterize the overall immunophenotypic architecture, PCA was performed on 11 immune cell parameters selected *a priori*: total T, B, and NK cells; CD4+ and CD8+ T cells; Th1, Th2, and Th17; total Tregs; and Treg subsets (CD45RA+ naïve and CD45RO+ memory Tregs). These parameters capture the principal compartments of innate and adaptive immunity implicated in RA pathogenesis. Variables were standardized (z-score transformation) prior to analysis. Components with eigenvalues exceeding the mean (Kaiser-Guttman criterion) were retained for visualization. PCA was conducted using the “vegan” package in R.

Moreover, LASSO regression (“glmnet” package) with 10-fold cross-validation was employed to identify the minimal set of immune markers optimally discriminating TN-RA from UA, with the optimal regularization parameter (λ) selected using the 1-standard-error rule. Model performance was comprehensively assessed by (i) discrimination (area under receiver operating characteristic curve with 95% confidence intervals, sensitivity, and specificity at optimal threshold); (ii) calibration (comparing predicted probabilities with observed proportions); (iii) clinical utility (decision curve analysis quantifying net benefit across risk thresholds); and (iv) internal validation (bootstrap resampling with B = 1000 to estimate optimism-corrected performance).

To address potential confounding, we performed multivariable logistic regression adjusting for age, sex, BMI, and disease duration. Further adjustment for RF titers, anti−CCP titers, ESR, and CRP was attempted but led to non−convergent models due to near−complete separation, reflecting collinearity between these serological markers and RA classification criteria.

## 3 Results

### 3.1 Clinical and demographic characteristics of patients in the retrospective study

**Table 1** describes the demographic characteristics, disease features, laboratory indicators, and current treatments of 132 RA patients (49 with TN-RA and 83 with TE-RA), 51 patients with UA, and 60 HCs. The average age of the RA group in this study was 57.10 ± 13.16 years for TN-RA and 57.75 ± 12.45 years for TE-RA. The HC group, with a mean age of 57.40 ± 5.54 years and 58.33% female participants, was matched for age and sex with the RA patients. The average age of the UA group was 44.61 ± 13.37 years, which is younger than that of the TN-RA and TE-RA groups. The duration of the disease in TE-RA was prolonged compared to TN-RA and UA. The inflammatory markers ESR, CRP, and neutrophil-to-lymphocyte ratio in RA patients were elevated compared to the UA group; however, no statistically significant difference was observed between the two RA groups. The hemoglobin concentration in the two RA groups was lower than that of the UA group. There were no significant differences in demographic characteristics, disease activity measures, or serological autoantibody levels (RF, anti-MCV, and anti-CCP antibodies) between the two groups of RA patients.

**Table 1:** Demographic and clinical characteristics of the study participants.

	TN-RA (n=49)	TE-RA (n=83)	UA (n=51)	HC (n=60)	*P*-value
Demographic					
Male, n (%)	10 (20.40%)	26 (31.30%)	15 (29.40%)	25 (41.67%)	0.122
Female, n (%)	39 (79.60%)	57 (68.70%)	36 (70.60%)	35 (58.33%)	
Age (years)	57.10 ± 13.16	57.75 ± 12.45 ^b***^	44.61 ± 13.37 ^c***^	57.40 ± 5.54^d***^	<0.001^***^
BMI (kg/m^2^)	21.30(23.88-24.92)	21.48(23.74-26.04)	22.27(24.56-27.34)	–	0.202
Disease duration (years)	0.50(1.00-6.00) ^a***^	4.00(8.00-14.00) ^b***^	0.50(3.00-7.00)	–	<0.001^***^
Lifestyle					
Smoking, n (%)	13 (26.50%)	17 (20.50%)	9 (17.60%)	–	0.538
Drinking, n (%)	4 (8.20%)	7 (8.40%)	6 (11.80%)	–	0.772
Laboratory characteristics					
ESR (mm/h)	19.00(45.00-66.00)	16.00(29.00-74.00) ^b***^	4.00(7.50-13.00) ^c***^	–	<0.001^***^
CRP (mg/ml)	3.31(9.16-24.67)	3.31(11.26-34.80) ^b***^	1.11(3.27-3.31) ^c***^	–	<0.001^***^
DAS-28- ESR	4.85 ± 1.66	4.99 ± 1.37	–	–	–
WBC (*10^9/L)	5.09(6.47-8.48)	5.03(6.39-8.77)	5.13(6.15-7.02)	–	0.166
LY (*10^9/L)	1.15(1.42-1.72)	1.05(1.39-1.93)	1.32(1.62-2.00)	–	0.193
Hb (g/L)	128.51 ± 17.27	127.51 ± 20.70 ^b***^	141.31 ± 19.03 ^c**^	–	<0.001^***^
PLT (*10^9/L)	234.00(275.00-344.00)	214.00(267.00-334.00)	212.00(240.50-302.00)	–	0.115
PLR	151.27(188.89-238.41)	132.08(188.71-251.89)	134.42(157.75-200.66) ^c*^	–	0.04^*^
NLR	2.34(3.10-4.37)	2.21(3.00-4.39) ^b**^	1.71(2.21-2.80) ^c**^	–	0.001^**^
PT (s)	11.33 ± 0.69	11.21 ± 0.67	11.01 ± 0.69	–	0.064
APTT (s)	28.89 ± 3.42	29.36 ± 3.76 ^b***^	27.23 ± 2.77	–	0.002^**^
D-dimer (ug/L)	89.00(258.00-544.00)	98.00(293.50-665.00) ^b***^	14.00(20.00-50.00) ^c***^	–	<0.001^***^
Fibrinogen (mmol/L)	3.20(3.84-4.40)	3.16(3.86-4.80) ^b***^	2.32(2.76-3.13) ^c***^	–	<0.001^***^
ALT (U/L)	11.20(18.10-28.60)	12.10(15.65-19.50) ^b***^	16.40(23.24-31.70) ^c*^	–	<0.001^***^
AST (U/L)	15.80(18.90-23.90)	16.80(19.00-24.30) ^b**^	19.30(22.85-27.60) ^c*^	–	0.004^**^
ALP (U/L)	77.00(87.00-105.00)	75.00(87.00-107.00) ^b**^	63.00(78.00-88.00) ^c*^	–	0.005^**^
GGT (U/L)	15.50(20.50-35.40)	14.80(20.80-28.50)	16.30(24.25-32.70)	–	0.412
ALB (g/L)	35.90(40.90-43.20)	37.40(39.95-42.20) ^b***^	42.20(45.60-47.40) ^c***^	–	<0.001^***^
BUN (mmol/L)	4.40(5.10-5.70)	4.40(5.50-6.50)	4.30(4.80-5.80)	–	0.117
Cr (μmol/L)	48.60(55.80-62.70)	48.70(57.70-67.70)	51.20(58.20-66.00)	–	0.719
RF (U/m L)	32.70(104.70-220.10)	35.10(203.55-300.00) ^b***^	0.00(0.00-21.50) ^c***^	–	<0.001^***^
Anti-CCP (U/mL)	186.77(495.95-500.00)	126.17(500.00-500.00) ^b***^	36.00(26.00-50.00) ^c***^	–	<0.001^***^
Anti- MCV (U/mL)	55.20(277.10-871.50)	32.00(233.15-879.10) ^b***^	0.00(0.00-0.00) ^c***^	–	<0.001^***^
APF, n (%)	31 (63.27%)	42 (50.60%) ^b***^	0 (0.00%) ^c***^	–	<0.001^***^
ANA, n (%)	22 (44.90%)	26 (31.30%) ^b***^	0 (0.00%) ^c***^	–	<0.001^***^
Medication					
NSAIDs, n (%)	2 (4.08%) ^a***^	28 (33.73%) ^b***^	0 (0.00%)	–	<0.001^***^
Prednisone, n (%)	0 (0.00%) ^a***^	34 (41.00%) ^b***^	0 (0.00%)	–	<0.001^***^
csDMARDs, n (%)	0 (0.00%) ^a***^	50 (60.20%) ^b***^	0 (0.00%)	–	<0.001^***^
MTX, n (%)	0 (0.00%) ^a***^	26 (31.30%) ^b***^	0 (0.00%)	–	<0.001^***^
LEF, n (%)	0 (0.00%) ^a***^	30 (36.10%) ^b***^	0 (0.00%)	–	<0.001^***^
bDMARDs, n (%)	0 (0.00%) ^a**^	11 (13.30%) ^b**^	0 (0.00%)	–	0.007^**^

Data are presented as mean ± standard deviation for normally distributed continuous variables, median (interquartile range) for non-normally distributed continuous variables, and number (percentage) for categorical variables. RA, rheumatoid arthritis; TN-RA, treatment-naïve RA; TE-RA, treatment-experienced RA; UA, undifferentiated arthritis; HC, healthy control; BMI, body mass index; ESR,erythrocyte sedimentation rate; CRP, C-reactive protein; DAS-28, Disease Activity Score 28; WBC, white blood cell; LY, lymphocyte; Hb, hemoglobin; PLT, platelet; PLR, platelet-to-lymphocyteratio; NLR, neutrophil-to-lymphocyte ratio; PT, prothrombin time; APTT, activated partial thromboplastin time; ALT, alanine transaminase; AST, aspartic transaminase; ALP, alkalinephosphatase; GGT, gamma-glutamyl transferase; ALB, albumin; BUN, blood urea nitrogen; Cr, creatinine; RF, rheumatoid factor; anti-CCP, anti-cyclic citrullinated peptide antibody; Anti-MCV, anti-mutated citrullinated vimentin; APF, antiperinuclear factor; ANA, antinuclear antibodies; NSAIDs, nonsteroidal anti-inflammatory drugs; csDMARDs, conventional synthetic disease-modifying anti-rheumatic drugs; MTX, methotrexate; LEF, leflunomide; bDMARDs, biological DMARDs.

The *p*-value column indicates the overall comparison across all three or four groups using a one-way ANOVA, Kruskal-Wallis H test, or chi-square test, as appropriate. Superscript letters denote pairwise comparisons: a, TN-RA vs. TE-RA; b, TE-RA vs. UA; c, UA vs. TN-RA; d, UA vs. HC. Asterisks indicate statistical significance (*p < 0.05, **p < 0.01, ***p < 0.001).

### 3.2 Immune landscape across disease states

We first performed unsupervised PCA on 11 predefined immune cell parameters to assess qualitative immune architecture across disease states. The first two components explained 57.75% of the total variance (PCA1, 45.87%; PCA2, 11.88%). The two-dimensional projection revealed substantial overlap among RA, UA, and HC groups, reflecting considerable inter-individual heterogeneity in peripheral immunophenotypes (**Supplementary Figure 2**). Variable loadings identified the regulatory-effector balance as the primary axis of variation, with Treg-related vectors opposing those of Th1, Th17, and CD8+ T cells.

### 3.3 Characteristics of peripheral blood lymphocyte profiles in RA and UA patients

**Figure 2:**
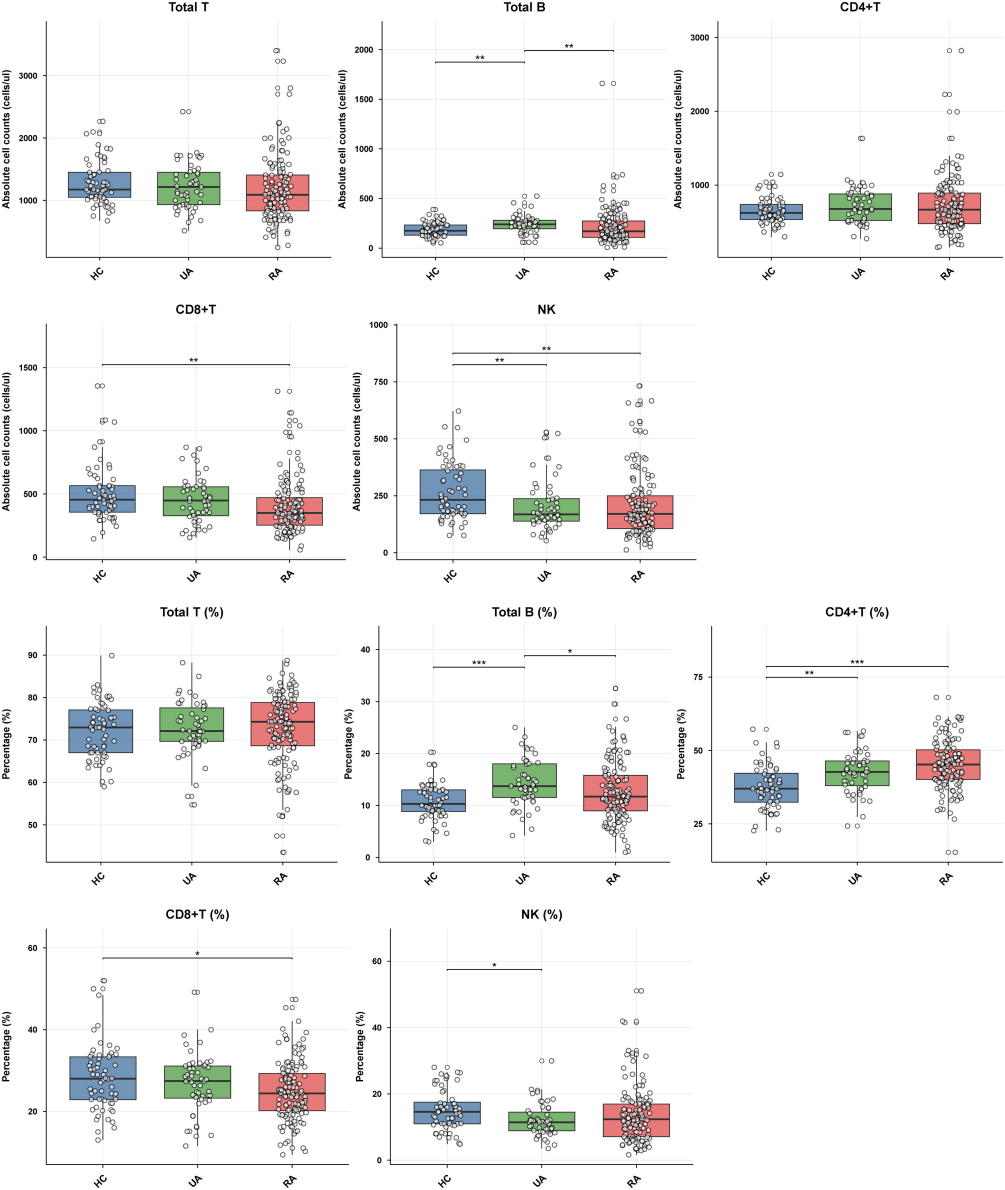
Comparison of peripheral lymphocyte levels among rheumatoid arthritis (RA), undifferentiated arthritis (UA), and healthy control (HC). T, T lymphocyte; B, B lymphocyte; NK, natural killer cell. (*p < 0.05, **p < 0.01, ***p < 0.001).

We quantified major lymphocyte populations in peripheral blood, including T cells (CD45+CD3+), B cells (CD45+CD3−CD19+), NK cells (CD45+CD3−CD16+CD56+), CD4+ T cells (CD45+CD3+CD4+), and CD8+ T cells (CD45+CD3+CD8+). There were no significant differences in the total number of T cells or CD4+ T cells between the RA, UA, and HC groups. There are more B cells in the UA group than in the RA and HC groups (*p* < 0.05), and there are also more NK cells in the UA and RA groups than in the HC group (*p* < 0.05). The absolute number of CD8+ T cells was significantly decreased in the RA group relative to the HC group [RA: 347.93 (252.83-459.73) cells/μL vs. HCs: 455.36 (355.39-566.48) cells/μL; *p* = 0.001] (**Supplementary Table 1**; **Figure 2**). Subsequently, a comparison was conducted among the four groups (TN-RA, TE-RA, UA, and HCs). The analysis revealed no statistically significant disparity in immune cell levels between the TN-RA and TE-RA groups. Furthermore, the total B-cell count in the UA group was predominantly higher than that of the TE-RA group, with no observed difference compared to the TN-RA group (**Supplementary Figure 3**).

### 3.4 Different polarization of the Th17/Treg axis in RA and UA

**Figure 3:**
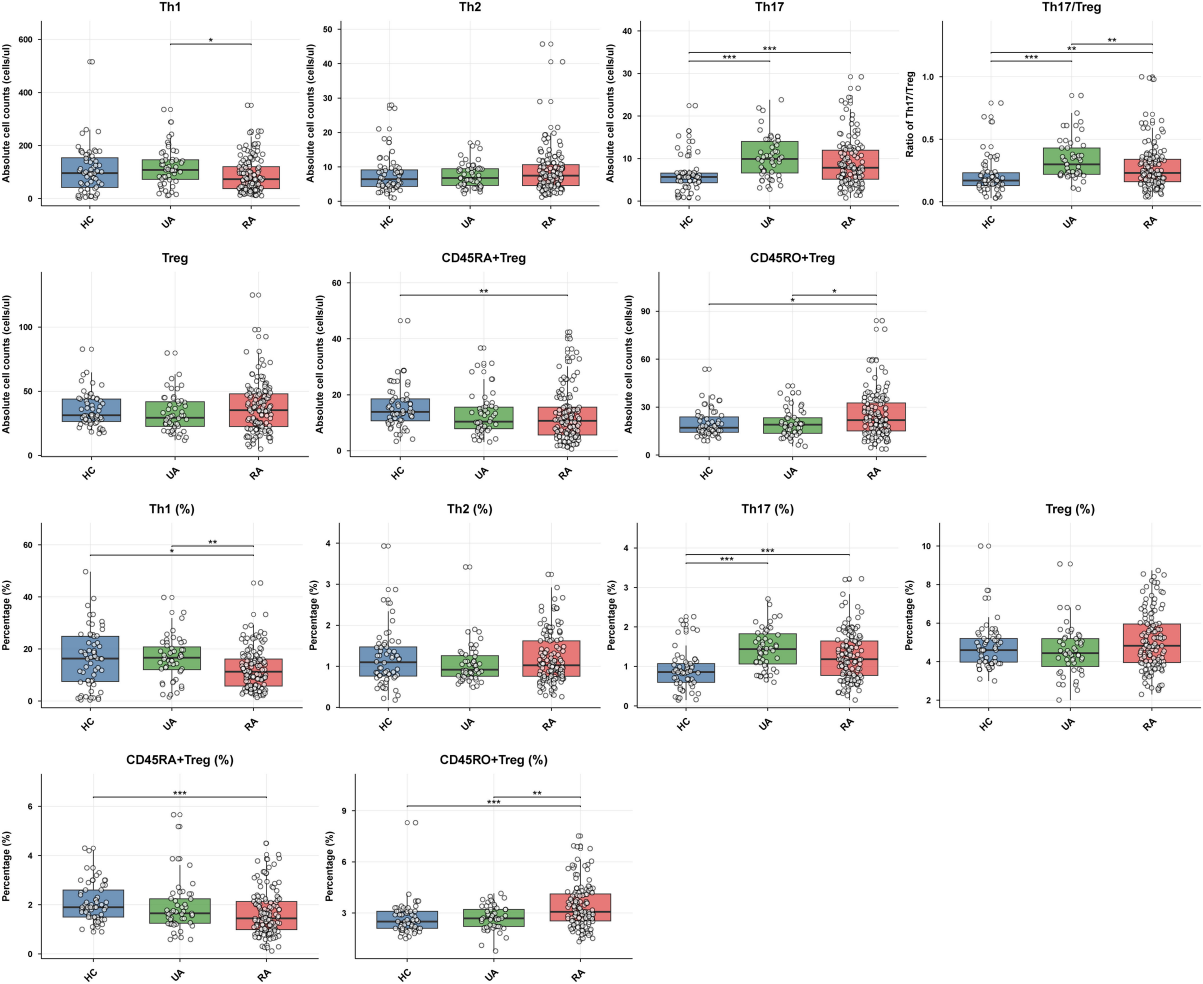
Comparison of peripheral CD4+ T cell and Treg subset levels among rheumatoid arthritis (RA), undifferentiated arthritis (UA), and healthy control (HC). Th1, T-helper 1 cells; Th2, T-helper 2 cells; Th17, T-helper 17 cells; Treg, regulatory T cells. (*p < 0.05, **p < 0.01, ***p < 0.001).

Our examination of CD4+ helper and regulatory subsets identified the critical anomaly. We observed that both RA and UA demonstrated an increased Th17/Treg ratio compared to HCs, with the Th17/Treg ratio in UA surpassing that in RA [RA: 0.23 (0.16-0.34) vs. UA: 0.30 (0.22-0.43); *p* = 0.003]. RA and UA exhibited an elevated absolute count and percentage of Th17 cells in comparison to HCs; however, no statistically significant difference was observed between UA and RA. Furthermore, while the overall Treg counts in RA patients did not significantly differ from those in HCs, the absolute count and percentage of CD45RA+ Tregs were diminished compared to HCs, whereas the absolute count and percentage of CD45RO+ Tregs were elevated relative to HCs. However, no differences were observed between CD45RA+ Tregs and CD45RO+ Tregs in both UA and HCs. Consequently, the Th17/Treg axis polarization differs between RA and UA. UA is characterized by Th17-driven inflammation with a higher Th17/Treg ratio and no compensatory regulatory response, suggesting an early inflammatory state. In RA, the lower Th17/Treg ratio appears to result from compensatory expansion of CD45RO+ memory Tregs (**Supplementary Table 1**; **Figure 3**).

**Figure 4:**
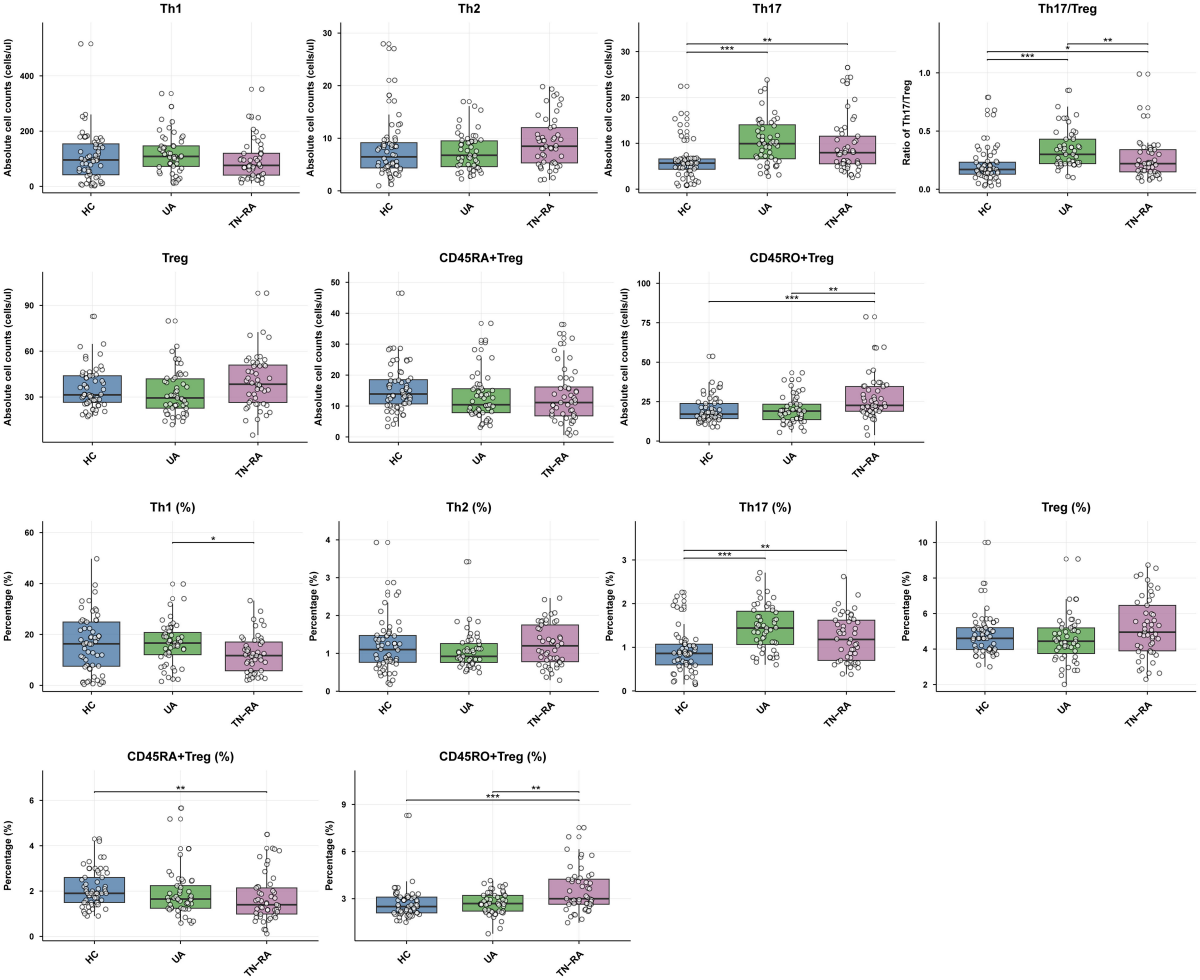
Comparison of peripheral CD4+ T cell and Treg subset levels among treatment-naïve RA (TN-RA), undifferentiated arthritis (UA), and healthy control (HC). Th1, T-helper 1 cells; Th2, T-helper 2 cells; Th17, T-helper 17 cells; Treg, regulatory T cells. (*p < 0.05, **p < 0.01, ***p < 0.001).

The analysis of CD4+ T and Treg cell subsets among the four groups (TN-RA, TE-RA, UA, and HCs) indicated no statistically significant difference in cell counts between the TN-RA and TE-RA groups. The number and percentage of Th1 cells in UA patients were much higher than in the TE-RA group, but there was no significant difference when compared to the TN-RA group. The Th17/Treg ratio in the UA group was much higher than in the TN-RA group, but there was no significant difference from the TE-RA group. Furthermore, the absolute number and percentage of CD45RO+ Tregs in the UA group were substantially lower than in the TN-RA group. Nonetheless, the quantities of CD45RA+ Tregs in the UA group did not significantly differ from those in the TN-RA and TE-RA groups. The TN-RA group showed a higher absolute count of CD45RO+ Tregs than the HC group, while the TE-RA group showed a lower absolute count of CD45RA+ Tregs. No statistically significant difference was seen in the overall count of Tregs and their subtypes between UA and HCs (**Figure 4**; **Supplementary Figure 3**).

### 3.5 Both UA and RA patients showed diminished IL-2 levels and elevated concentrations of other cytokines

**Figure 5:**
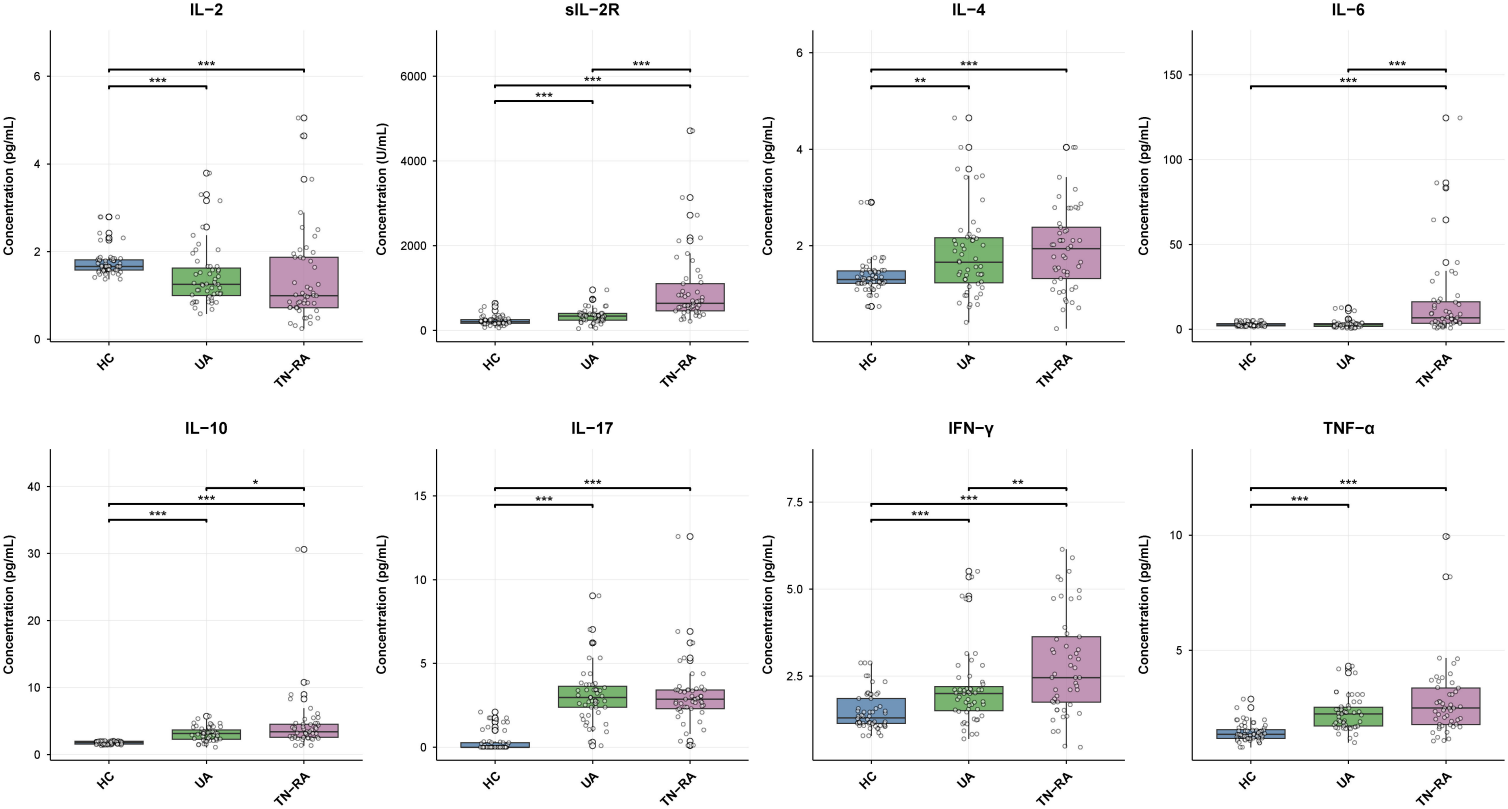
The level of various serum cytokines in treatment-naïve RA (TN-RA), undifferentiated arthritis (UA), and healthy control (HC). IL, interleukin; sIL-2R, soluble interleukin-2 receptor; IFN-γ, interferon-γ; TNF-α, tumor necrosis factor-α. (*p < 0.05, **p < 0.01, ***p < 0.001).

We focused on the levels of different blood cytokines in UA, TN-RA, TE-RA, and HCs. The IL-2 levels were lower in the UA and RA groups than in the HCs. The levels of sIL-2R, IL-4, IL-6, IL-10, IL-17, IFN-γ, and TNF-α were significantly higher in the two RA groups than in the HCs. However, in the UA group, all cytokines increased except for IL-6. The levels of these cytokines were not statistically different between the TN-RA and TE-RA groups. Furthermore, the levels of sIL-2R, IL-6, and IFN-γ in TN-RA and TE-RA are higher than those in UA, while the levels of IL-10 and TNF-α are higher than those in UA (**Supplementary Figure 4, 5**). Compared with UA, TN−RA patients had significantly higher serum levels of sIL−2R, IL−6, IL−10, and IFN-γ (all *p* < 0.05), reflecting a more pronounced proinflammatory state in TN−RA (**Figure 5**).

### 3.6 Analysis of the correlation between general clinical markers, peripheral lymphocyte cell counts, and cytokine levels in the TN-RA and UA groups

**Figure 6:**
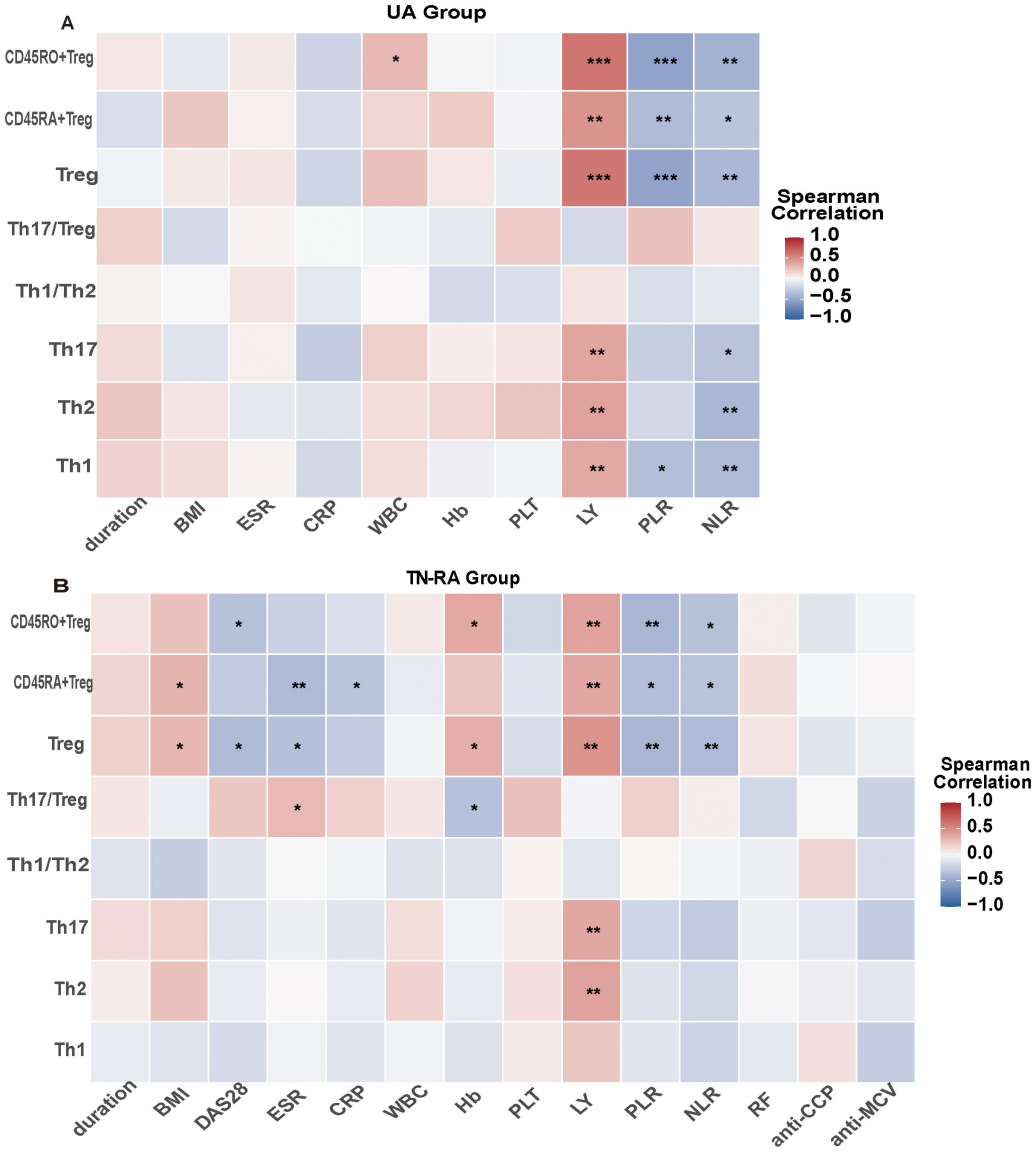
Heat map of correlation between CD4+ T cell counts and general clinical indicators in UA and TN-RA. TN-RA, treatment-naïve RA; UA, undifferentiated arthritis. (*p < 0.05, **p < 0.01, ***p < 0.001).

**Figure 7:**
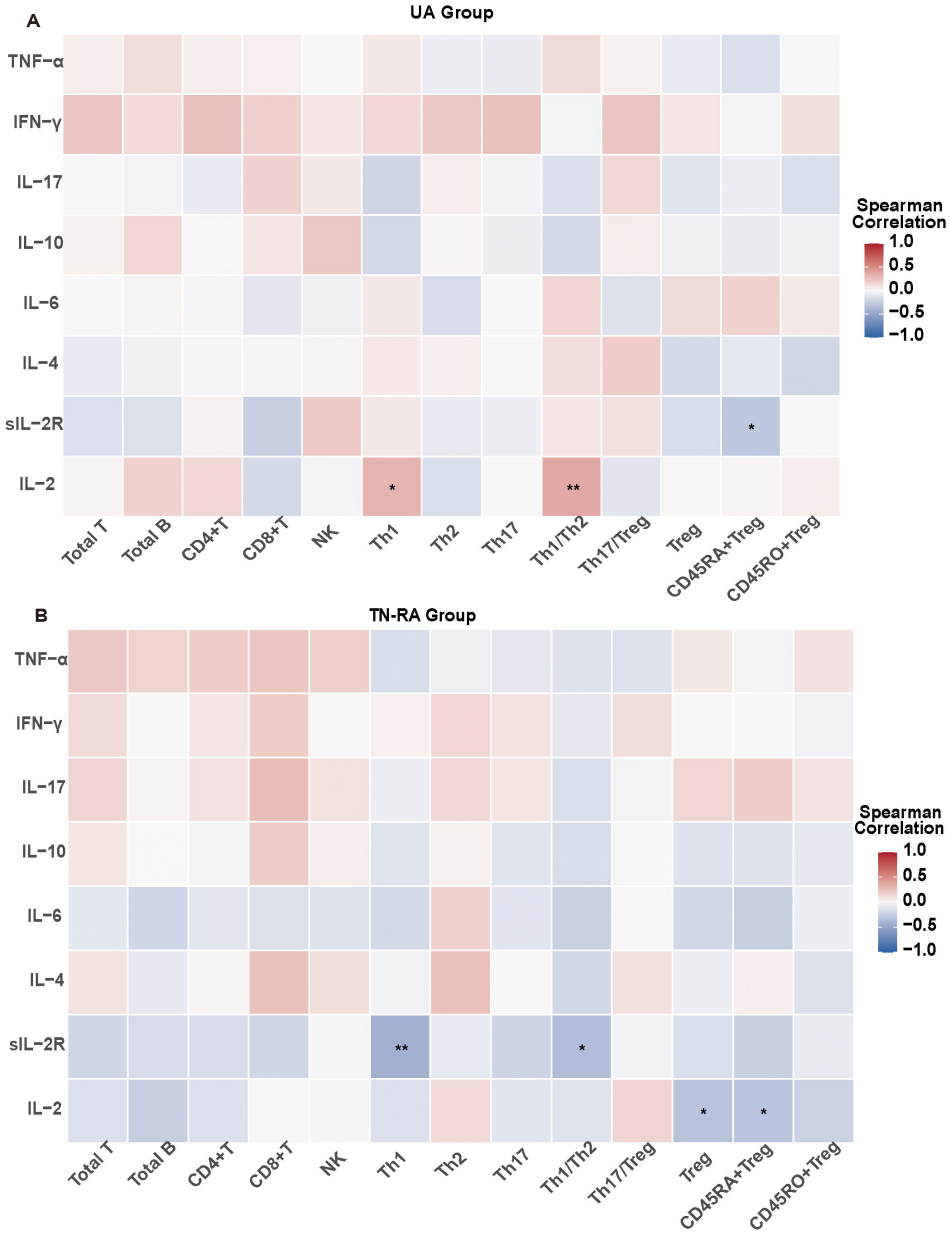
Heat map of correlation between cytokine levels and peripheral lymphocyte counts in UA and TN-RA. TN-RA, treatment-naïve RA; UA, undifferentiated arthritis. (*p < 0.05, **p < 0.01).

In the UA group, correlation analysis indicated that general clinical indicators did not significantly correlate with CD4+ T cell subsets; however, IL-2 concentrations demonstrated a positive correlation with Th1 cell counts, while sIL-2R concentrations exhibited a negative correlation with CD45RA+ Treg counts (**Figures 6A**, **7A**). In the TN-RA group, DAS28 demonstrated an inverse correlation with total Treg and CD45RO+ Treg counts. The levels of ESR and CRP showed a negative relationship with the number of CD45RA+ Tregs. Moreover, IL-2 levels in TN-RA demonstrated a negative correlation with Th1 cell counts, whereas sIL-2R levels indicated a negative correlation with Treg and CD45RA+ Treg counts (**Figures 6B**, **7B**). Furthermore, CRP levels correlated positively with IL-6 and TNF-α levels in UA. IL-2 concentrations negatively correlated with RA duration. In RA, IL-6 concentrations correlated with ESR and CRP levels, while sIL-2R concentrations correlated with DAS28, ESR, CRP, and anti-MCV levels (**Supplementary Figure 6**).

### 3.7 An eight-immune feature risk effectively distinguished RA from UA

**Figure 8:**
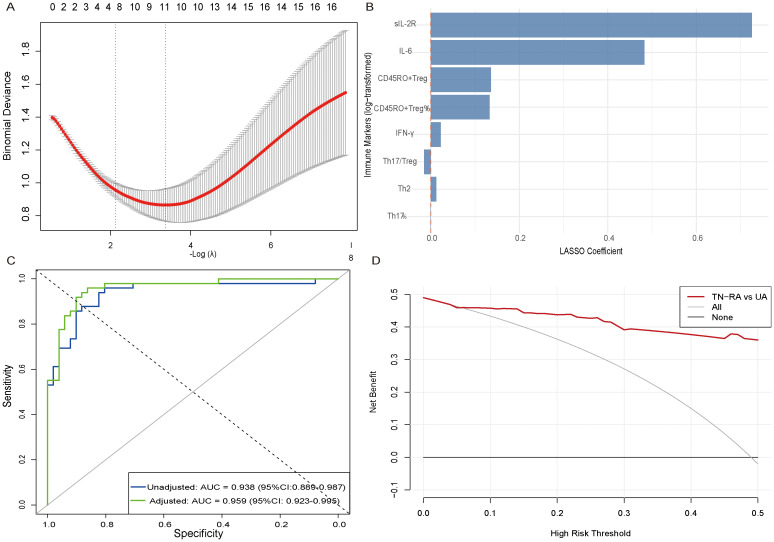
Performance of the LASSO-derived immune signature for distinguishing TN-RA from UA. **(A)** LASSO regression with 10-fold cross-validation for selection of the optimal regularization parameter (λ). **(B)** LASSO coefficients of the eight selected immune markers. **(C)** Receiver operating characteristic (ROC) analysis of LASSO-derived immune markers for distinguishing TN-RA from UA. The adjusted model was adjusted for age, sex, BMI, and disease duration. **(D)** Decision curve analysis evaluating clinical utility. Positive net benefit across threshold probabilities 0.1–0.5 supports clinical utility for guiding treatment decisions in patients with early inflammatory arthritis. TN-RA, treatment-naïve rheumatoid arthritis; UA, undifferentiated arthritis; AUC, area under the curve; CI, confidence interval.

LASSO regression with 10-fold cross-validation generated an eight-marker immunological profile that effectively discriminated TN-RA from UA at initial presentation (**Figure 8A**). The ideal regularization parameter (λ) was determined using the 1-standard-error approach, resulting in a concise model with eight non-zero coefficients (**Figure 8B**). The selected markers included sIL-2R, IL-6, CD45RO+ Tregs, CD45RO+ Treg percentage, IFN-γ, Th17/Treg ratio, Th2, and Th17 percentage. sIL-2R (coefficient = 0.725) and IL-6 (coefficient = 0.482) emerged as the most significant contributors, aligning with the exhaustion of IL-2 signaling and systemic inflammation as characteristics of established RA, whereas memory Treg markers (memory Tregs and memory Treg percentage) exhibited positive coefficients, suggesting compensatory expansion in TN-RA (**Figure 8B**; **Supplementary Figure 7**). After adjustment for age, sex, BMI, and disease duration, the eight−feature immune signature maintained strong discriminatory performance (adjusted AUC = 0.959, 95% CI: 0.923–0.995) (**Figure 8C**). sIL−2R and IL−6 remained the strongest predictors in the adjusted model (**Supplementary Table 2**). Decision curve analysis revealed a favorable net benefit within clinically significant risk thresholds (0.1-0.5), endorsing its clinical relevance in informing diagnostic decisions (**Figure 8D**). Calibration curves demonstrated good agreement between predicted and observed probabilities (**Supplementary Figure 8**).

## 4 Discussion

Analysis of immunophenotypes revealed differences in Treg compartment dynamics between RA and UA. UA was characterized by Th17-driven inflammation without a compensatory regulatory response, a pattern consistent with an early inflammatory state. In RA, the lower Th17/Treg ratio appeared to arise from compensatory expansion of CD45RO+ memory Tregs rather than diminished Th17 activity, suggesting a state of perturbed Treg homeostasis during disease evolution. We developed a derivation-stage classifier consisting of eight immunological markers that shows potential for differentiating RA from UA. These findings align with a model in which established RA is characterized by reduced IL-2 bioavailability and compensatory memory Treg expansion, in contrast to Th17-predominant inflammation in UA. By focusing on therapy-naïve patients, we eliminated treatment-related confounding and identified intrinsic disease-associated immunophenotypes. Sensitivity analyses adjusting for age, sex, BMI, and disease duration confirmed that the immune signature maintained its discriminatory performance.

The imbalance between Th17 and Tregs plays an important role in RA. Th17 cells primarily promote inflammation by the secretion of IL-17, thus accelerating the onset of autoimmune disorders (25). Conversely, Tregs facilitate the anti-inflammatory response by the secretion of IL-10 and TGF-β, therefore sustaining autoimmune tolerance (10). The elevated levels of Th17 cells observed in both UA and RA indicate a shared high-inflammatory state in these diseases. CD45RA+ naïve Tregs and CD45RO+ memory Tregs represent distinct Treg subsets: the former are mostly thymic-derived and require peripheral antigen encounter for full activation, whereas the latter are generated upon antigenic stimulation, exhibit superior tissue tropism and migratory capacity, and persist independently of ongoing antigen exposure (11, 26–28). Previous research has reported Th17 expansion concurrent with Treg reduction in established RA (13, 14, 29). However, our study found no significant difference in total Treg counts between RA patients and HCs. A subsequent Treg subset analysis revealed a significant finding: TN-RA patients exhibited a marked increase in memory Treg counts. The negative correlation between memory Tregs and DAS28 indicates that a loss or compromised maintenance of this regulatory subset may facilitate disease exacerbation. Memory Tregs initially demonstrate augmented inhibitory function (26). The increase in memory Tregs in RA may suggest a phenotypic adaptation to chronic inflammation; whether this constitutes a functionally competent compensatory response or a marker of dysregulated homeostasis remains undetermined. Further investigation is needed to clarify the suppressive role of memory Tregs in RA and how it evolves with disease progression.

Our findings indicated that both UA and RA patients exhibited diminished levels of IL-2 and elevated levels of sIL-2R, consistent with reduced IL-2 bioavailability. IL-2 signaling is essential for the function, stability, and survival of Tregs, which have high-affinity IL-2 receptors, enabling them to respond to minimal doses of IL-2 (16, 30). The elevated memory Tregs in RA may prove functionally impaired if IL-2 deprivation is confirmed, which is essential for their survival and the inhibition of other cell proliferation (31). Nonetheless, Tregs cannot autonomously produce IL-2 and rely totally on an external source of IL-2 (15). The negative correlation between IL-2 concentration and RA disease duration indicates a potential progressive exhaustion of IL-2 production or a shift in the cellular source of IL-2 over time, which may compromise immune homeostasis and contribute to a persistent dysregulated state. Consequently, low-dose IL-2 therapy may contribute to the prevention of RA development through the restoration of Treg function. A study reported that low-dose IL-2 preferentially stimulates the proliferation of endogenous circulating Tregs, potentially providing therapeutic benefits in systemic lupus erythematosus (30, 31). The significant positive association of sIL-2R with clinical disease activity (DAS28), acute-phase reactants (ESR, CRP), and autoantibody (anti-MCV) levels in rheumatoid arthritis underscores its function as a reliable biomarker of systemic immune activation and disease severity. sIL-2R, a component of the IL-2Rα chain, is acknowledged as a crucial serological marker of activated T cells entering the bloodstream subsequent to immunological activation (32, 33). In accordance with our findings, serum sIL-2R levels have been identified as a disease marker for RA and correlate with disease progression, indicating that a reduction in sIL-2R levels may arise from joint improvement (34).

The LASSO-selected immune features reveal distinct yet mechanistically interconnected pathways underlying early RA pathogenesis. Notably, sIL-2R and IL-6 emerged as the strongest predictive contributors, reflecting the central roles of T-cell activation dynamics and systemic inflammation in disease initiation (20, 35, 36). Elevated sIL-2R levels indicate persistent T-cell turnover and IL-2 signaling exhaustion (35), whereas IL-6 drives acute-phase responses and synovial proliferation (37). Concurrently, the inclusion of Th17 cells and IFN-γ underscores the pathogenic contribution of type 17 immunity to tissue inflammation (38, 39), while the altered representation of CD45RO+ Tregs points to disrupted regulatory homeostasis during disease progression. These findings corroborate prior reports identifying IL-6 and sIL-2R as robust biomarkers of RA activity (40, 41). Moreover, the co-selection of memory Treg markers (CD45RO+ Tregs and CD45RO+ Treg percentage) alongside Th17-related parameters suggests a compensatory regulatory response that characterizes established RA, in contrast to the Th17-predominant immune imbalance observed in UA—highlighting the immunological transition from early inflammation to chronic autoimmunity.

Several limitations should be noted in our study. First, the cross-sectional design compares established TN-RA with concurrent UA at a single time point; consequently, our model serves as a derivation-stage classifier rather than a diagnostic tool for real-world consecutive populations, and the observed discrimination may be exaggerated. External and preferably prospective validation is required. Whether UA patients with RA-like immune profiles progress to fulfill RA criteria remains unknown. Second, single-center derivation and modest sample size necessitate external validation in independent cohorts. Finally, the absence of functional assays—specifically Treg suppressive capacity assessments and *in situ* synovial tissue localization—limits mechanistic interpretation of the observed phenotypic associations.

## 5 Conclusion

In conclusion, we have developed a derivation-stage immune signature using machine learning that shows potential for discriminating TN-RA from UA at initial presentation. The IL-2–Treg axis perturbation, reflected by sIL-2R and memory Treg markers, represents a candidate pathophysiological distinction between these disease states. This objective immune-based approach may support clinical decision-making when conventional criteria are inconclusive, potentially facilitating timely management of early RA. Future studies should externally validate the signature in independent (preferably prospective) cohorts and assess its real-world impact on diagnostic confidence, treatment decisions, and long-term outcomes.

## Data Availability

The raw data supporting the conclusions of this article will be made available by the authors, without undue reservation.
