# Supplementary material for: Soluble IL-2 receptor and memory Treg profiles differentiate early rheumatoid arthritis from undifferentiated arthritis at initial presentation
Source: Front Immunol. 2026 Jun 9;17:1844268. doi: 10.3389/fimmu.2026.1844268 (PMC13286901; doi:10.3389/fimmu.2026.1844268)
Supplement: Supplementary file 1 [file Table1.docx]

Soluble IL-2 Receptor and Memory Treg Profiles Differentiate Early Rheumatoid Arthritis from Undifferentiated Arthritis at Initial Presentation

Supplementary Material

# Supplementary Figures and Tables

## Supplementary Figures


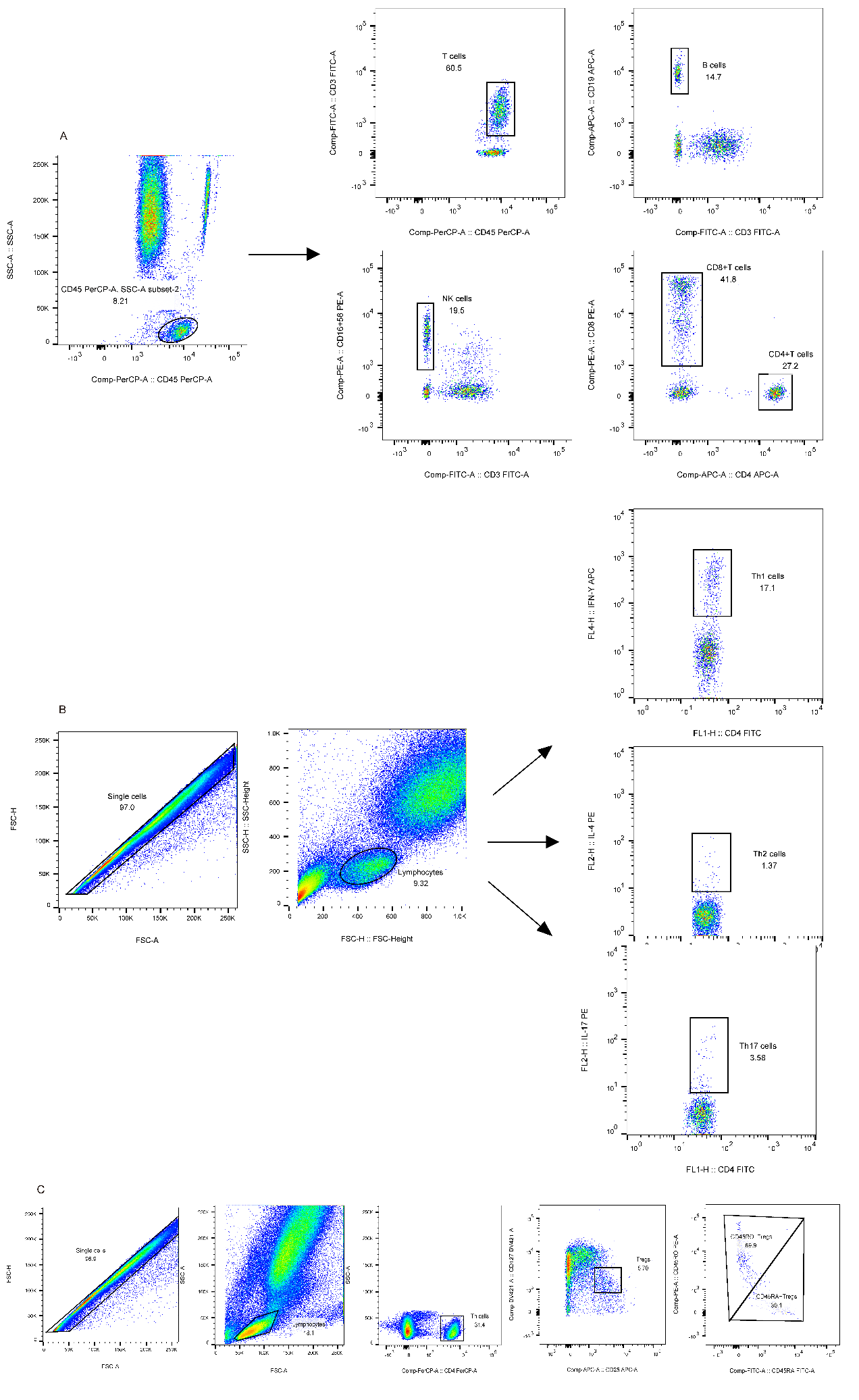


**Supplementary Figure 1** Gating strategy for flow cytometric analysis. **(A)** Sequential gating of peripheral blood lymphocytes. Lymphocytes were first identified by FSC-A/SSC-A, followed by singlet discrimination (FSC-A/FSC-H). T cells: CD45+CD3+; B cells: CD45+CD3−CD19+; NK cells: CD45+CD3−CD16+CD56+; CD4+ T cells: CD45+CD3+CD4+; CD8+ T cells: CD45+CD3+CD8+. **(B)** CD4+ T cell intracellular cytokine staining following PMA/ionomycin stimulation. Th1: CD4+IFN-γ+; Th2: CD4+IL-4+; Th17: CD4+IL-17A+. **(C)** Treg phenotyping. Tregs were identified as CD4+CD25+CD127low/−, with further subdivision into CD45RA+ naïve Tregs and CD45RO+ memory Tregs.


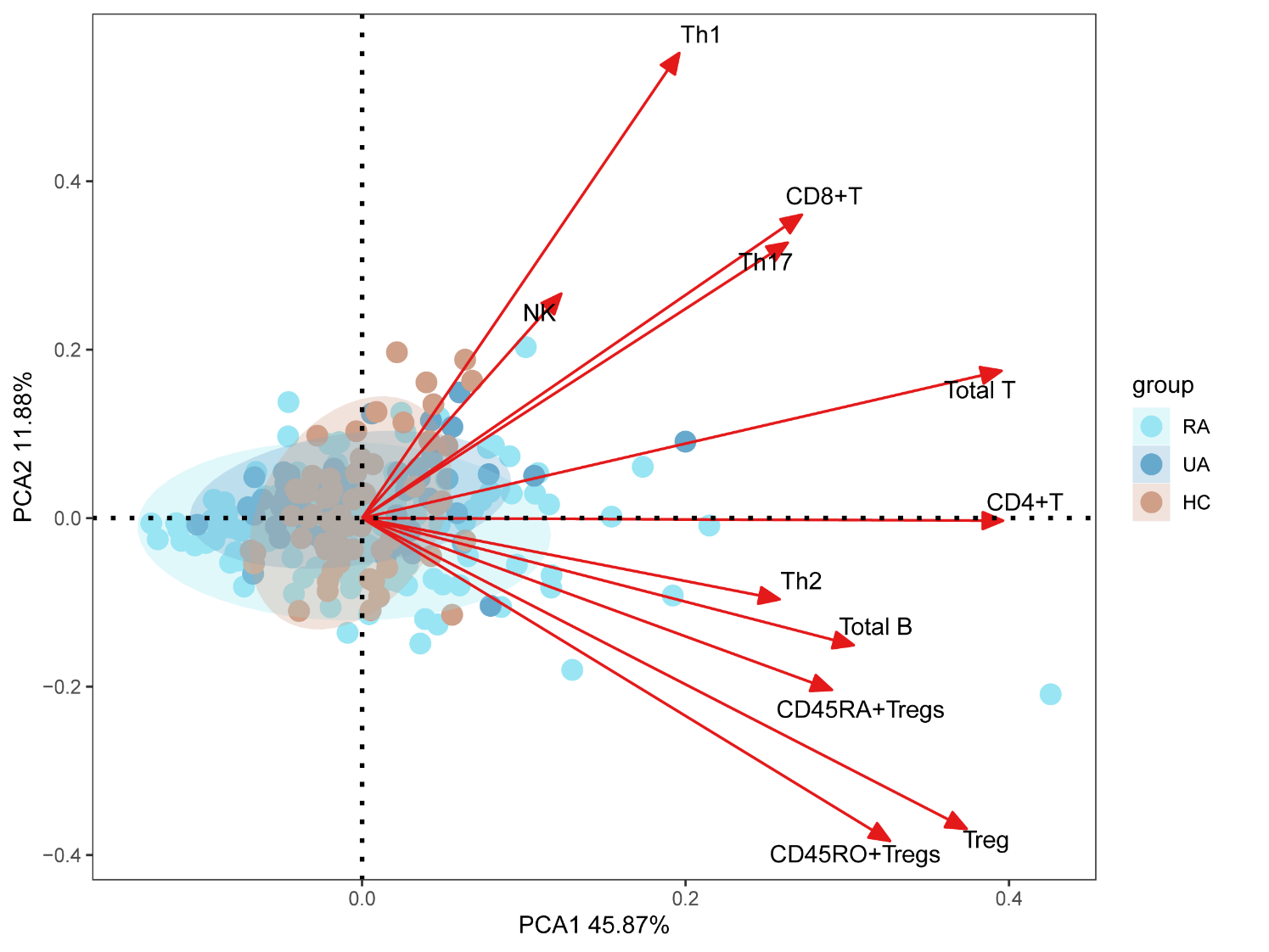


**Supplementary Figure 2** Principal component analysis (PCA) of immune profiles in RA, UA, and HC. PCA1 and PCA2 explain 45.87% and 11.88% of the total variance, respectively. RA: rheumatoid arthritis; UA: undifferentiated arthritis; HC: healthy controls.


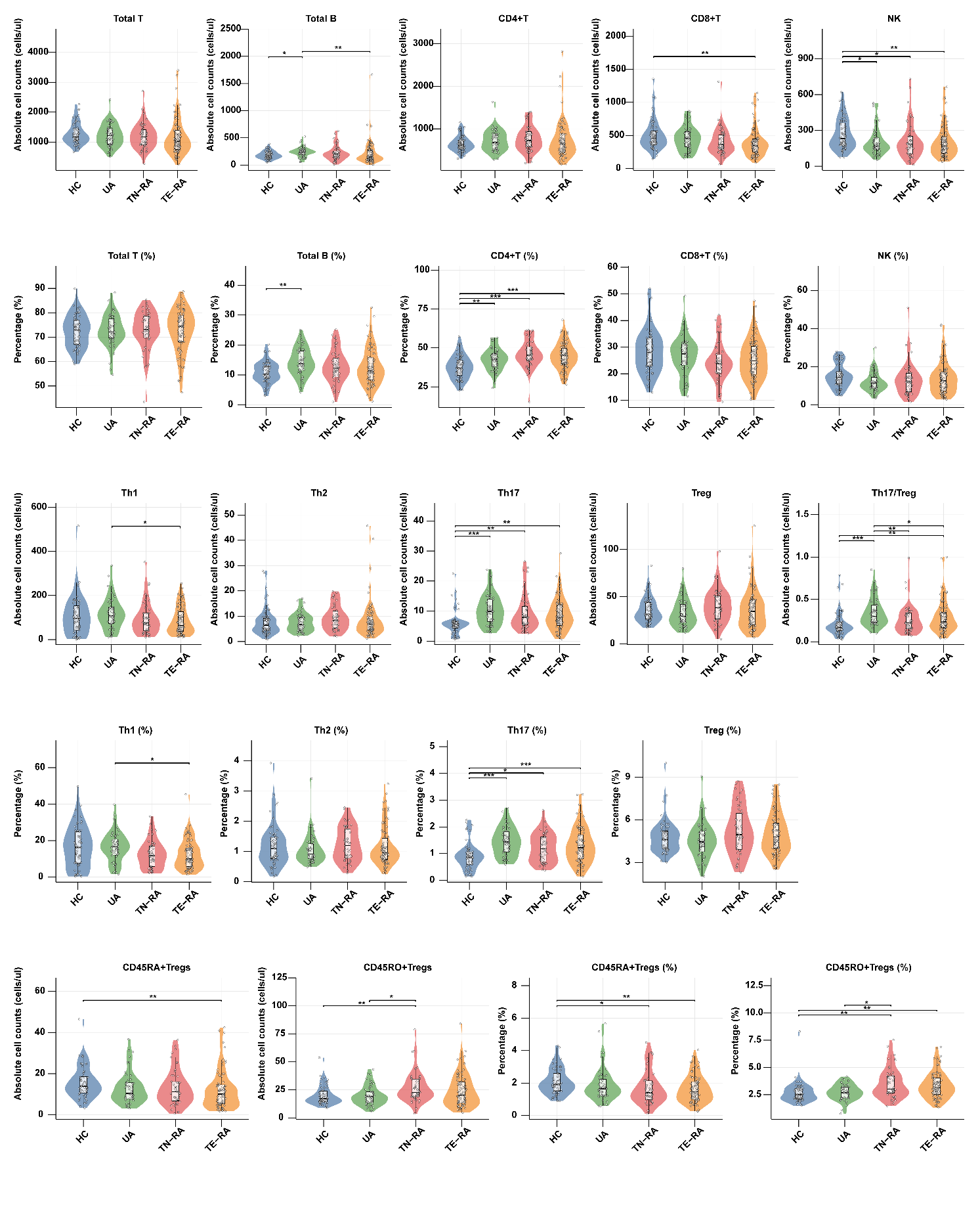


**Supplementary Figure 3** Levels of peripheral blood lymphocytes, CD4+ T and Treg cell subsets in patients with TN-RA, TE-RA, UA, and HC. RA: rheumatoid arthritis; TN-RA: treatment-naïve RA; TE-RA: treatment-experienced RA; UA: undifferentiated arthritis; HC: healthy controls; Treg: regulatory T cells. (**p* < 0.05, ***p* < 0.01, ****p* < 0.001)


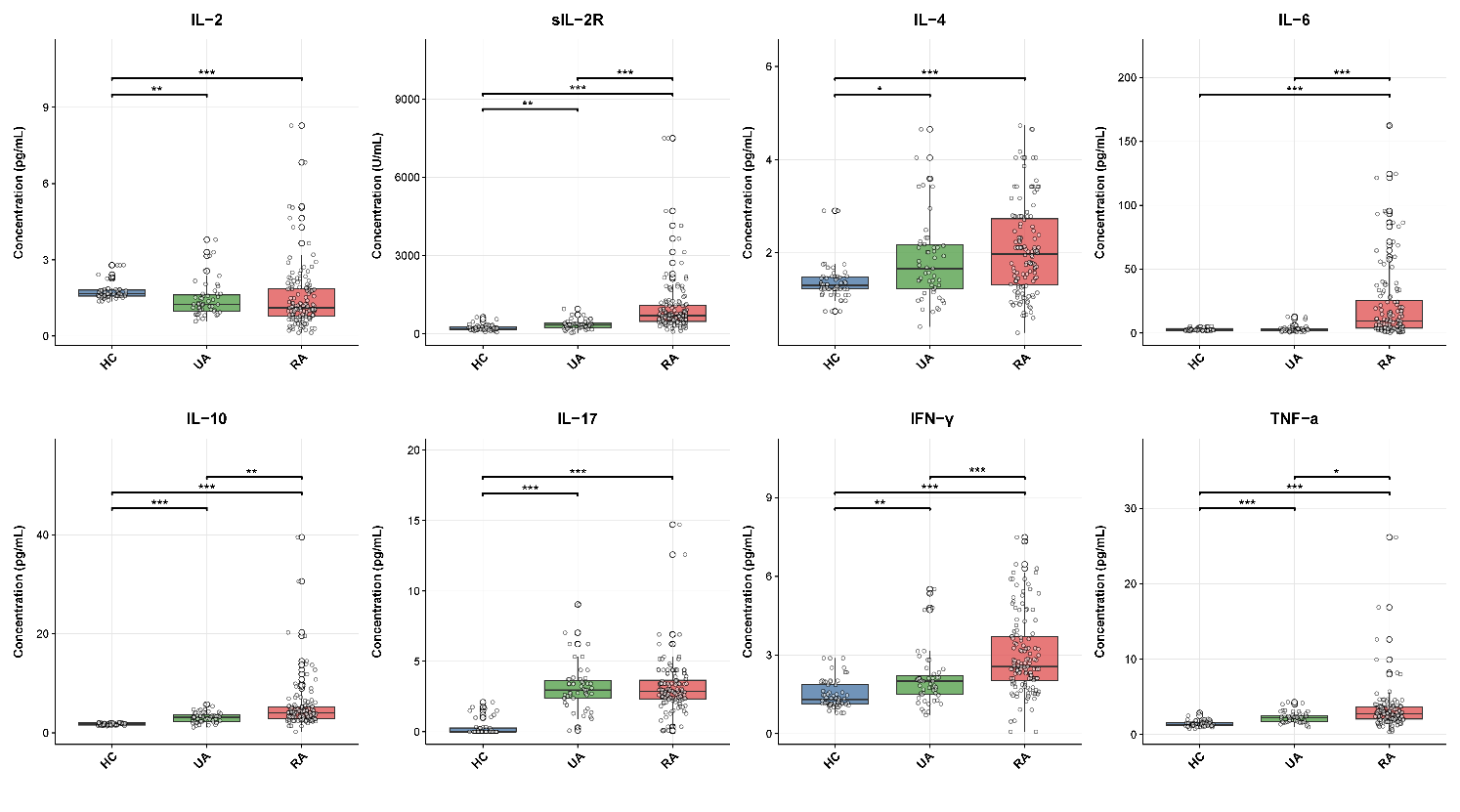


**Supplementary Figure 4** The level of various serum cytokines in RA, UA, and HCs. UA: undifferentiated arthritis; HCs: healthy controls. IL: interleukin; sIL-2R: soluble interleukin-2 receptor; IFN-γ: interferon-γ; TNF-α: tumor necrosis factor-α. (**p* < 0.05, ***p* < 0.01, ****p* < 0.001).


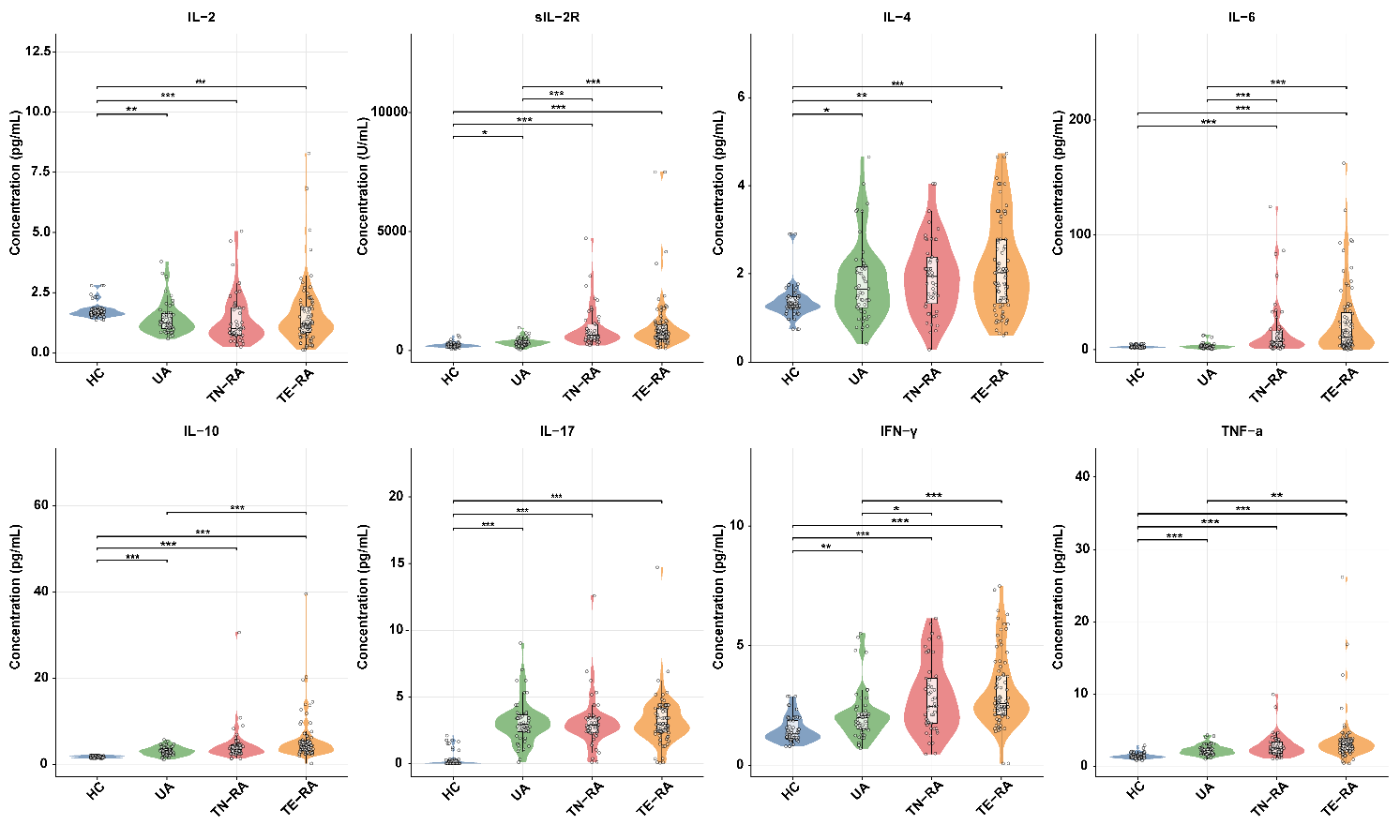


**Supplementary Figure 5** The level of various serum cytokines in TN-RA, TE-RA, UA, and HCs. TN-RA: Treatment-naïve RA; TE-RA: Treatment-experienced RA; UA: undifferentiated arthritis; HCs: healthy controls. IL: interleukin; sIL-2R: soluble interleukin-2 receptor; IFN-γ: interferon-γ; TNF-α: tumor necrosis factor-α. (**p* < 0.05, ***p* < 0.01, ****p* < 0.001).


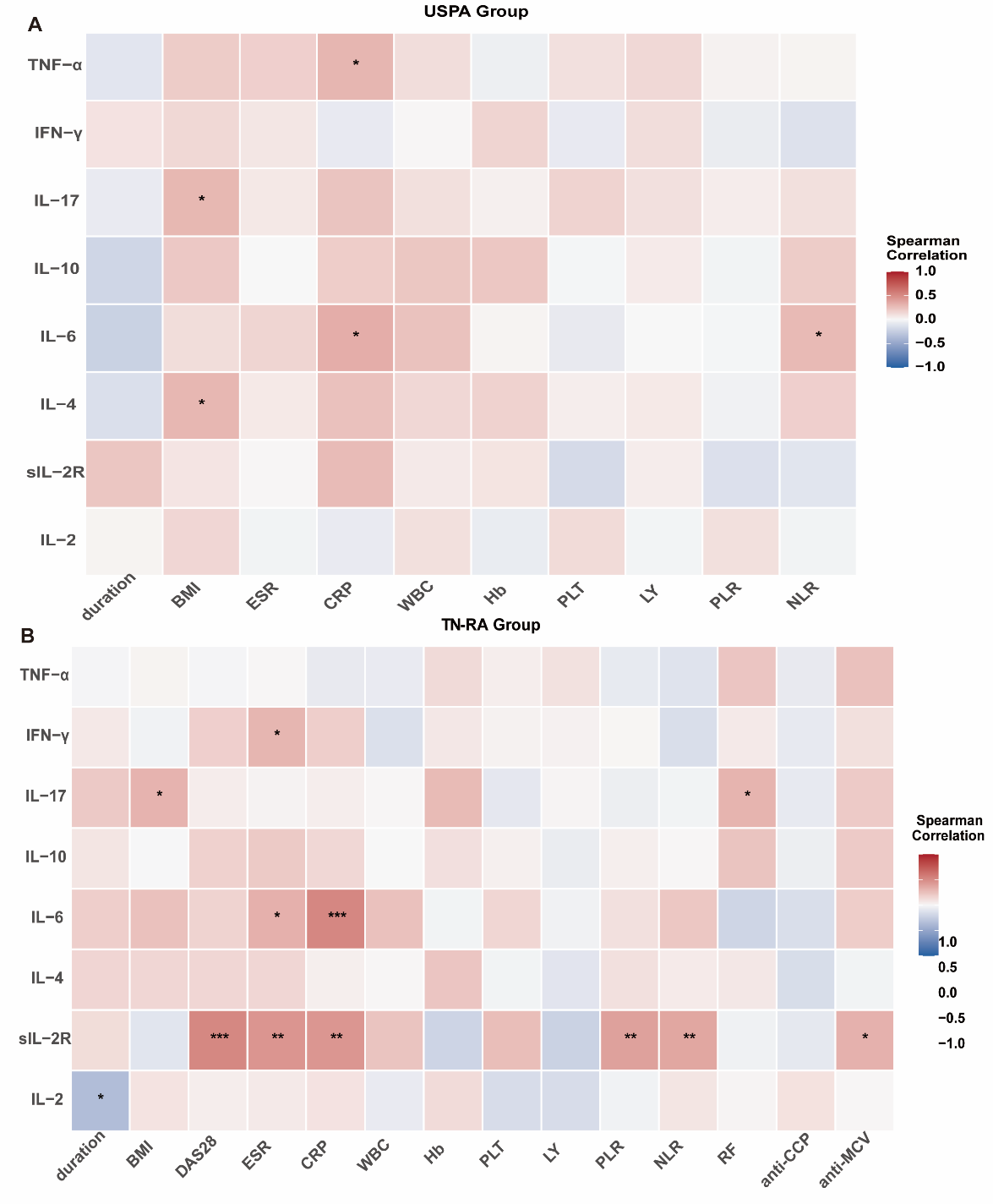


**Supplementary Figure 6** Correlation heat map of cytokine levels and general clinical indicators in patients with TN-RA and UA groups. **A**: Heat map of correlation between cytokine levels and general clinical indicators in UA. **B**: Heat map of correlation between cytokine levels and general clinical indicators in TN-RA. TN-RA: Treatment-naïve RA; UA: undifferentiated arthritis. (**p* < 0.05, ***p* < 0.01, ****p* < 0.001).


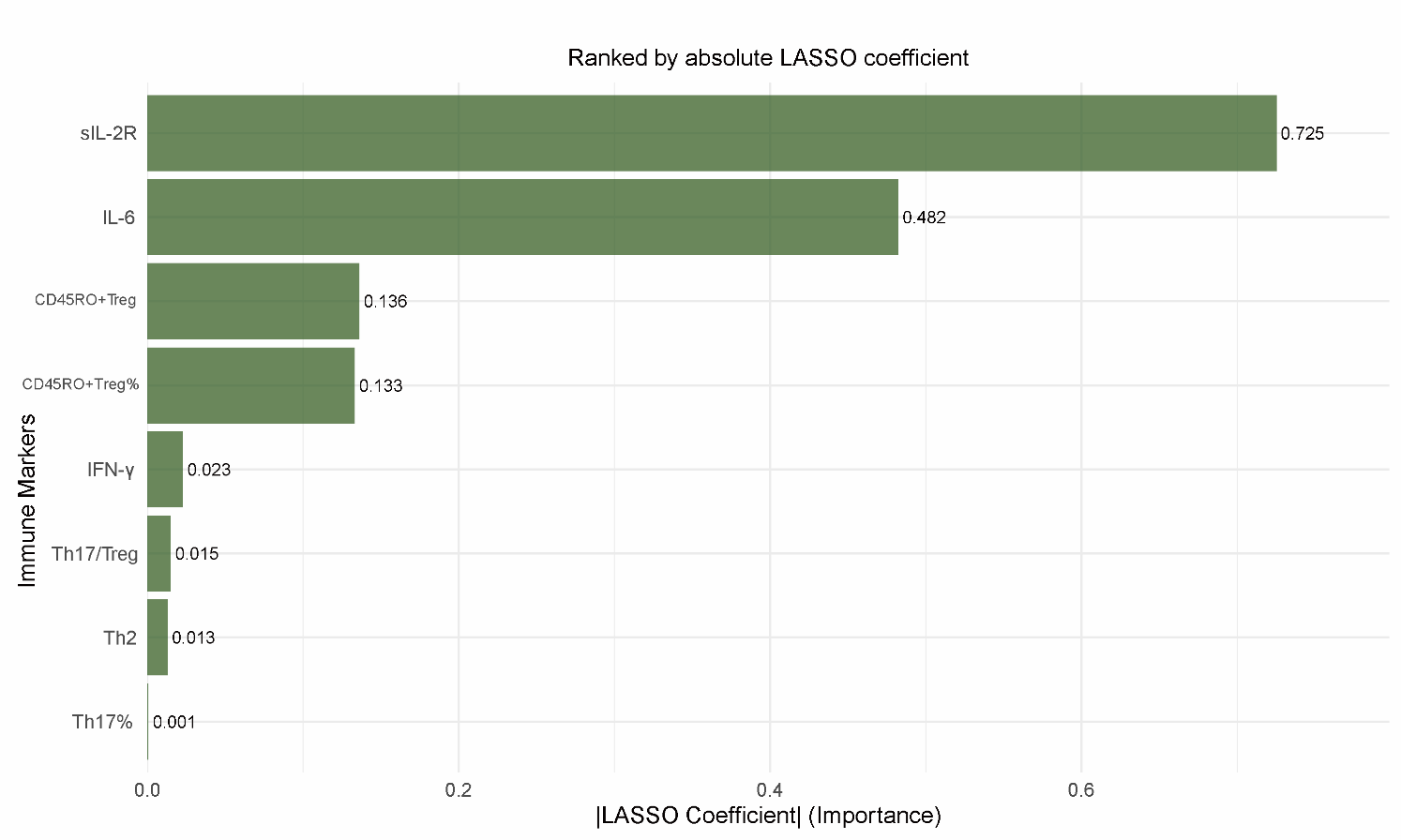


**Supplementary Figure 7** Feature importance ranking for the immune diagnostic classifier. Immune markers ranked by absolute LASSO coefficient magnitude (|β|). sIL-2R (|β| = 0.725) and IL-6 (|β| = 0.482) were the two most important contributors, together accounting for the majority of discriminative power.


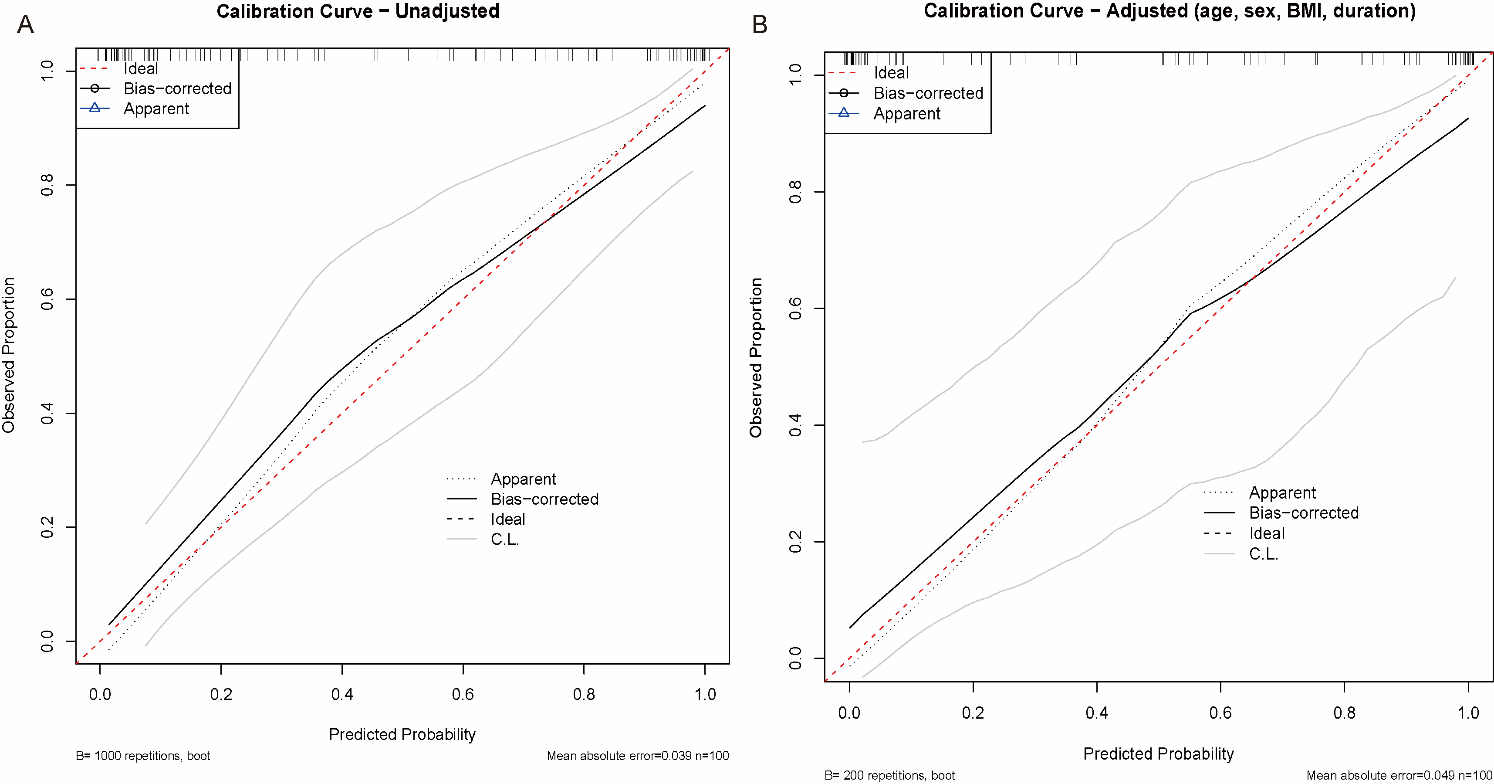


**Supplementary Figure 8** Calibration of the immune signature risk prediction model. Calibration plot comparing predicted probabilities (x-axis) against observed proportions of TN-RA (y-axis). The dotted line represents apparent calibration, the solid line represents bias-corrected calibration (bootstrap, B=1000), and the dashed line represents ideal calibration. The grey lines indicate 95% confidence intervals. Mean absolute error = 0.039. The close agreement between bias-corrected and ideal calibration demonstrates good model calibration, supporting the reliability of predicted probabilities for clinical risk stratification. TN-RA: treatment-naïve rheumatoid arthritis.

## Supplementary Tables

**Supplementary Table 1** Absolute counts and proportions of lymphocytes, CD4+ T, and Treg subsets in the peripheral blood among HCs, UA, and RA.

|  | HCs (n=60) | UA (n=51) | RA (n=132) | *p*-value | *p*-value,  HCs vs. UA | *p*-value,  UA vs. RA | *p*-value,  HCs vs. RA |
| --- | --- | --- | --- | --- | --- | --- | --- |
| Total T (cells/μL) ^b^ | 1173.98(1044.13-1451.17) | 1215.28(932.52-1450.43) | 1089.13(832.92-1404.59) | 0.059 | - | - | - |
| T% ^b^ | 72.96(67.00-77.15) | 72.11(69.66-77.57) | 74.28(68.57-78.75) | 0.483 | - | - | - |
| Total B (cells/μL) ^b^ | 174.94(130.50-234.03) | 240.01(195.08-280.09) | 169.71(108.06-270.95) | 0.005** | 0.017* | 0.007** | 1.000 |
| B% ^b^ | 10.30(8.75-13.00) | 13.74(11.54-18.01) | 11.73(8.96-15.85) | <0.001*** | <0.001*** | 0.033* | 0.113 |
| CD4+T (cells/μL) ^b^ | 624.00(534.57-745.57) | 676.65(519.91-880.14) | 669.26(478.00-891.59) | 0.441 | - | - | - |
| CD4+T% ^a^ | 37.53±7.53 | 42.64±7.10 | 45.21±8.39 | <0.001*** | 00.003** | 0.149 | <0.001*** |
| CD8+T (cells/μL) ^b^ | 455.36(355.39-566.48) | 447.60(327.30-556.11) | 347.93(252.83-459.73) | 0.001** | 1.000 | 0.057 | 0.001** |
| CD8+T% ^b^ | 28.02(22.82-33.38) | 27.45(23.24-31.12) | 24.36(20.17-29.30) | 0.017* | 1.000 | 0.195 | 0.026* |
| CD4+T/CD8+T ^b^ | 1.35(1.04-1.95) | 1.55(1.23-2.08) | 1.87(1.46-2.49) | <0.001*** | 0.157 | 0.138 | <0.001*** |
| NK (cells/μL) ^b^ | 231.66(170.80-368.20) | 168.00(138.65-237.31) | 171.89(106.04-250.78) | 0.001** | 0.024* | 1.000 | 0.001** |
| NK% ^b^ | 14.59(10.98-17.77) | 11.44(8.87-14.48) | 12.37(7.19-16.95) | 0.020* | 0.027* | 1.000 | 0.063 |
| Th1 (cells/μL) ^b^ | 95.79(40.67-155.90) | 108.27(72.29-146.58) | 73.76(37.86-120.32) | 0.021* | 0.472 | 0.018* | 0.712 |
| Th1% ^b^ | 16.32(7.44-24.93) | 16.62(12.06-20.77) | 11.30(5.74-16.14) | 0.003** | 1.000 | 0.009** | 0.05 |
| Th2 (cells/μL) ^b^ | 6.46(4.34-9.32) | 6.77(4.61-9.50) | 7.49(4.64-10.69) | 0.573 | - | - | - |
| Th2% ^b^ | 1.10(0.76-1.48) | 0.92(0.76-1.26) | 1.02(0.76-1.63) | 0.538 | - | - | - |
| Th1/Th2 ^b^ | 14.42(5.65-21.46) | 17.02(9.95-23.79) | 9.52(5.46-18.42) | 0.013* | 0.485 | 0.011* | 0.514 |
| Th17 (cells/μL) ^b^ | 5.67(4.25-6.60) | 9.93(6.62-14.03) | 7.92(5.36-12.00) | <0.001*** | <0.001*** | 0.113 | <0.001*** |
| Th17% ^b^ | 0.86(0.60-1.07) | 1.44(1.07-1.83) | 1.18(0.77-1.65) | <0.001*** | <0.001*** | 0.114 | <0.001*** |
| Treg (cells/μL) ^b^ | 31.39(26.34-43.90) | 29.39(22.70-41.86) | 35.36(22.90-48.13) | 0.333 | - | - | - |
| Treg % ^b^ | 4.60(3.95-5.20) | 4.44(3.75-5.19) | 4.83(3.95-5.97) | 0.124 | - | - | - |
| Th17/Treg ^b^ | 0.17(0.13-0.24) | 0.30(0.22-0.43) | 0.23(0.16-0.34) | <0.001*** | <0.001*** | 0.003** | 0.005** |
| CD45RA+Treg (cells/μL) ^b^ | 13.89(10.57-18.68) | 10.42(7.86-15.59) | 11.01(5.70-15.61) | 0.003** | 0.126 | 1.000 | 0.002** |
| CD45RA+Treg % ^b^ | 1.90(1.50-2.60) | 1.65(1.25-2.24) | 1.44(0.99-2.11) | 0.001** | 0.205 | 0.406 | <0.001*** |
| CD45RO+Treg (cells/μL) ^b^ | 17.11(14.23-23.86) | 18.97(13.59-23.35) | 21.92(15.12-32.94) | 0.016* | 1.000 | 0.085 | 0.045* |
| CD45RO+Treg %^b^ | 2.50(2.10-3.10) | 2.69(2.21-3.21) | 3.09(2.55-4.15) | <0.001*** | 1.000 | 0.008** | <0.001*** |

Abbreviations: HC: Healthy control; UA: undifferentiated arthritis; RA: rheumatoid arthritis; T: T lymphocyte; B: B lymphocyte; NK: natural killer cell; Th1: T-helper 1 cells; Th2: T-helper 2 cells; Th17: T-helper 17 cells; Treg: regulatory T cells.

**^a^Results are expressed as the mean ± standard deviation.**

**^b^Results are expressed as the median and 25th and 75th percentiles.**

(**p* <0.05, ***p* <0.01, ****p* <0.001)

**Supplementary Table 2** Associations of LASSO-selected immune markers with TN-RA versus UA status.

| **Variable** | **Unadjusted model** | | | **Adjusted model*** | | |
| --- | --- | --- | --- | --- | --- | --- |
|  | **β** | OR(95%CI) | *p*-value | **β** | OR(95%CI) | *p*-value |
| sIL-2R | 2.360 | 10.59 (3.08-36.43) | **<0.001** | 3.013 | 20.35 (4.03-102.76) | **<0.001** |
| IL-6 | 1.747 | 5.74 (1.66-19.84) | **0.006** | 2.367 | 10.67 (2.21-51.62) | **0.003** |
| IFN-γ | 0.338 | 1.40 (0.62-3.16) | 0.415 | 0.337 | 1.40 (0.49-3.99) | 0.527 |
| Th2 | 0.895 | 2.45 (0.75-8.00) | 0.138 | 1.138 | 3.12 (0.77-12.66) | 0.111 |
| Th17% | -0.991 | 0.37 (0.05-2.98) | 0.351 | -1.093 | 0.34 (0.02-4.73) | 0.418 |
| Th17/Treg | 0.355 | 1.43 (0.36-5.63) | 0.613 | 0.306 | 1.36 (0.25-7.35) | 0.723 |
| CD45RO+Treg | 0.225 | 1.25 (0.38-4.12) | 0.710 | 0.521 | 1.68 (0.39-7.31) | 0.487 |
| CD45RO+Treg% | 1.069 | 2.91 (0.57-14.79) | 0.197 | 0.811 | 2.25 (0.34-14.86) | 0.400 |

**Notes**: Immune markers were selected by LASSO regression. *Adjusted for age, sex, BMI, and disease duration. Attempts to additionally adjust for RF, anti-CCP titers and inflammatory markers resulted in model non-convergence due to complete separation with RA diagnosis. OR, odds ratio; CI, confidence interval; sIL-2R, soluble interleukin-2 receptor; IL-6, interleukin-6; IFN-γ, interferon-gamma; Th, T helper; Treg, induced regulatory T cell. Significant ***P*** values (<0.05) are shown in bold.
